# Sawfishes and Other Elasmobranch Assemblages from the Mio-Pliocene of the South Caribbean (Urumaco Sequence, Northwestern Venezuela)

**DOI:** 10.1371/journal.pone.0139230

**Published:** 2015-10-21

**Authors:** Jorge D. Carrillo-Briceño, Erin Maxwell, Orangel A. Aguilera, Rodolfo Sánchez, Marcelo R. Sánchez-Villagra

**Affiliations:** 1 Paleontological Institute and Museum, University of Zurich, Zürich, Switzerland; 2 Staatliches Museum für Naturkunde, Stuttgart, Germany; 3 Universidade Federal Fluminense, Instituto de Biologia, Campus do Valonguinho, Outeiro São João Batista, Niterói, Rio de Janeiro, Brasil; 4 Museo Paleontológico de Urumaco, Urumaco, estado Falcón, Venezuela; New York Institute of Technology College of Osteopathic Medicine, UNITED STATES

## Abstract

The Urumaco stratigraphic sequence, western Venezuela, preserves a variety of paleoenvironments that include terrestrial, riverine, lacustrine and marine facies. A wide range of fossil vertebrates associated with these facies supports the hypothesis of an estuary in that geographic area connected with a hydrographic system that flowed from western Amazonia up to the Proto-Caribbean Sea during the Miocene. Here the elasmobranch assemblages of the middle Miocene to middle Pliocene section of the Urumaco sequence (Socorro, Urumaco and Codore formations) are described. Based on new findings, we document at least 21 taxa of the Lamniformes, Carcharhiniformes, Myliobatiformes and Rajiformes, and describe a new carcharhiniform species (†*Carcharhinus caquetius* sp. nov.). Moreover, the Urumaco Formation has a high number of well-preserved fossil *Pristis* rostra, for which we provide a detailed taxonomic revision, and referral in the context of the global Miocene record of *Pristis* as well as extant species. Using the habitat preference of the living representatives, we hypothesize that the fossil chondrichthyan assemblages from the Urumaco sequence are evidence for marine shallow waters and estuarine habitats.

## Introduction

The Caribbean Sea today is environmentally stable and ecologically complex, and it is characterized by high fish diversity [1]. However, the origin of this regional diversity is still somewhat problematic. In particular, Neogene chondrichthyan faunas in the Caribbean region are still little known in comparison with other regions as Europe and North America (e.g. [2]), although several previous works from diverse sedimentary basins exist (e.g. [3–24]) documenting the paleodiversity of Caribbean faunas before and after the complete closure of the Central American Seaway [25].

During the Miocene, northern Venezuela was close to the gateway between the Atlantic and Pacific Oceans prior to the definitive closure of the Panamanian Isthmus [25], and so faunal changes in this area provide an important perspective on changes in the Caribbean region as a whole. Neogene elasmobranchs from the Miocene of Venezuela have been described previously (e.g., [3, 15, 17, 20, 26]), and here we present new data on the elasmobranchs from the Urumaco sequence of northern Venezuela.

The Urumaco stratigraphic sequence is composed of seven geological units [27], represented by diverse paleoenvironment facies including marine, estuarine, riverine, lacustrine and terrestrial [27, 28]. Throughout the entire section [27], the lithology varies between more terrestrially influenced beds such as coal seams, and marine-influenced facies including sandstones, limestones and shales. The Urumaco sequence exhibits the most diverse vertebrate fauna from the Neogene of the southern Caribbean, including marine, estuarine, and freshwater fishes, freshwater and marine turtles and crocodilians, terrestrial and aquatic/semiaquatic mammals, and birds (e.g., [29– 32]). The stratigraphic sequence and the associated fauna provide unequivocal evidence of a marine coastal/estuarine environment, heavily influenced by a complex hydrographic system that flowed from western Amazonia to the proto-Caribbean Sea during the Miocene (e.g. [27, 31, 33–38]). More than 20 years of paleontological expeditions to the Urumaco region made available the collection of elasmobranch assemblages reported here. We provide a taxonomic revision of the elasmobranch fauna from the Socorro, Urumaco and Codore formations and discuss the paleoenvironmental implications. Importantly, we include new shark and ray occurrence data for Tropical America, and we provide a special detailed taxonomic revision of the specimens of *Pristis* “sawfishes” (Pristidae) found in these assemblages.

## Material and Methods

**Fig 1:**
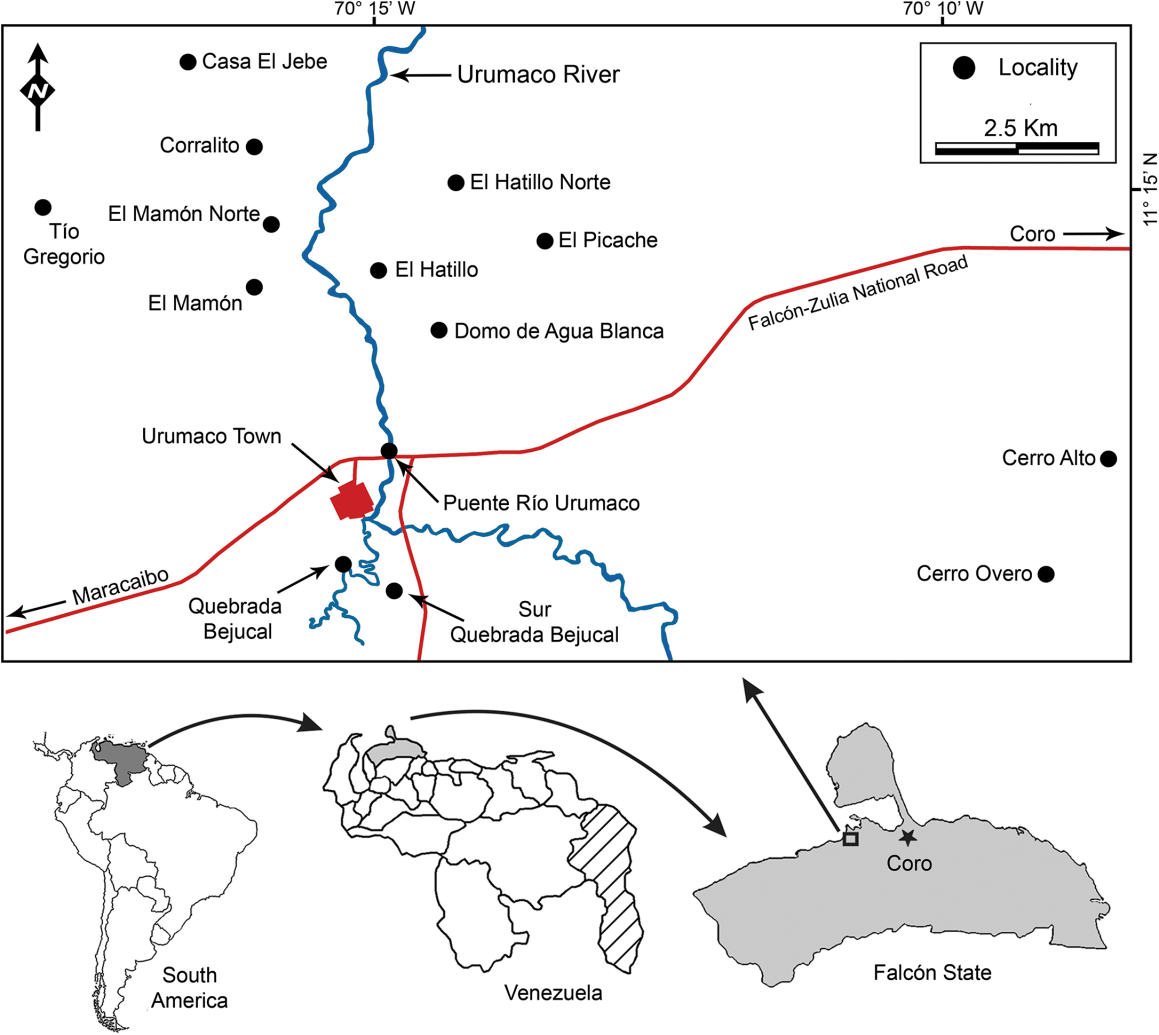
Locality map. Fossiliferous localities of the Urumaco sequence.

The fossil elasmobranch fauna from the Urumaco sequence presented here consists of 1234 specimens, and comes from 14 localities of the Socorro, Urumaco and Codore formations (Fig 1, S1 Table, S1 and S2 Appendix). Bulk samples of 10 kg each were collected on the outcrop and the sediment was screen washed using 0.5 mm open mesh. The specimens were sorted using a stereomicroscope. Large specimens were surface-collected from the outcrop. The material was collected by the authors and other collaborators since 1993 during several expeditions. The specimens are deposited in the paleontological collections of the Alcaldía Bolivariana de Urumaco (AMU-CURS), Centro de Investigaciones Antropológicas, Arqueológicas, Paleontológicas of the Universidad Experimental Francisco de Miranda (CIAAP, UNEFM-PF), and the Museo de Ciencias de Caracas (MCNC). The systematics for fossil and recent taxa follows Compagno [39] and Cappetta [2], with the exception of the extinct genus *Carcharocles* Jordan and Hannibal [40], whose assignment here has continued the discussion presented by Pimiento et al. [21]; the dental terminology follows Cappetta [2]. Terminology of the internal pristids rostral cavities follows Wueringer et al. [41]. Taxonomic identification included an extensive bibliographical review and comparative studies with fossil and extant specimens from the following collections: Fossil vertebrate section of the Museum für Naturkunde in Berlin (MB), Museo Nacional de Historia Natural de Santiago (SGO-PV) in Chile, Museu Paraense Emilio Goeldi (MPEG-V), Brazil; Natural History Museum of Basel (NMB), Switzerland; Palaeontological Institute and Museum at the University of Zurich (PIMUZ) Switzerland; René Kindlimann (private collection), Switzerland; Smithsonian Tropical Research Institute, Panama (STRI-PPP-T). We gathered habitat information of all taxa with living representatives using Compagno [42, 43], Compagno et al. [44], Musick et al. [45], Voigt and Weber [46], and the FishBase website (http://www.fishbase.org). The S2 Table is based on Aguilera [19], Aguilera and Lundberg [20], Lundberg et al. [47], Aguilera and Marceniuk [48], and Aguilera et al. [31].

Measurements for *Pristis* rostra follow the recommendations and methods proposed by Whitty et al. [49]. Rostral spine counts for extant species were obtained from the literature, as well as from examination of comparative material housed at the Museum of Natural History Vienna and the Zoological Museum of the University of Zurich. Rostral cavities in the fossil pristids were visible due to pre-existing breaks.

### Nomenclatural Acts

The electronic edition of this article conforms to the requirements of the amended International Code of Zoological Nomenclature, and hence the new names contained herein are available under that Code from the electronic edition of this article. This published work and the nomenclatural acts it contains have been registered in ZooBank, the online registration system for the ICZN. The ZooBank LSIDs (Life Science Identifiers) can be resolved and the associated information viewed through any standard web browser by appending the LSID to the prefix "http://zoobank.org/". The LSID for this publication is: urn:lsid:zoobank.org:pub: urn:lsid:zoobank.org:pub:8A290D4C-7169-4300-A15C-70942B19D153. The electronic edition of this work was published in a journal with an ISSN, and has been archived and is available from the following digital repositories: PubMed Central, LOCKSS.

## Geological Context

### Socorro Formation (Middle Miocene)

**Fig 2:**
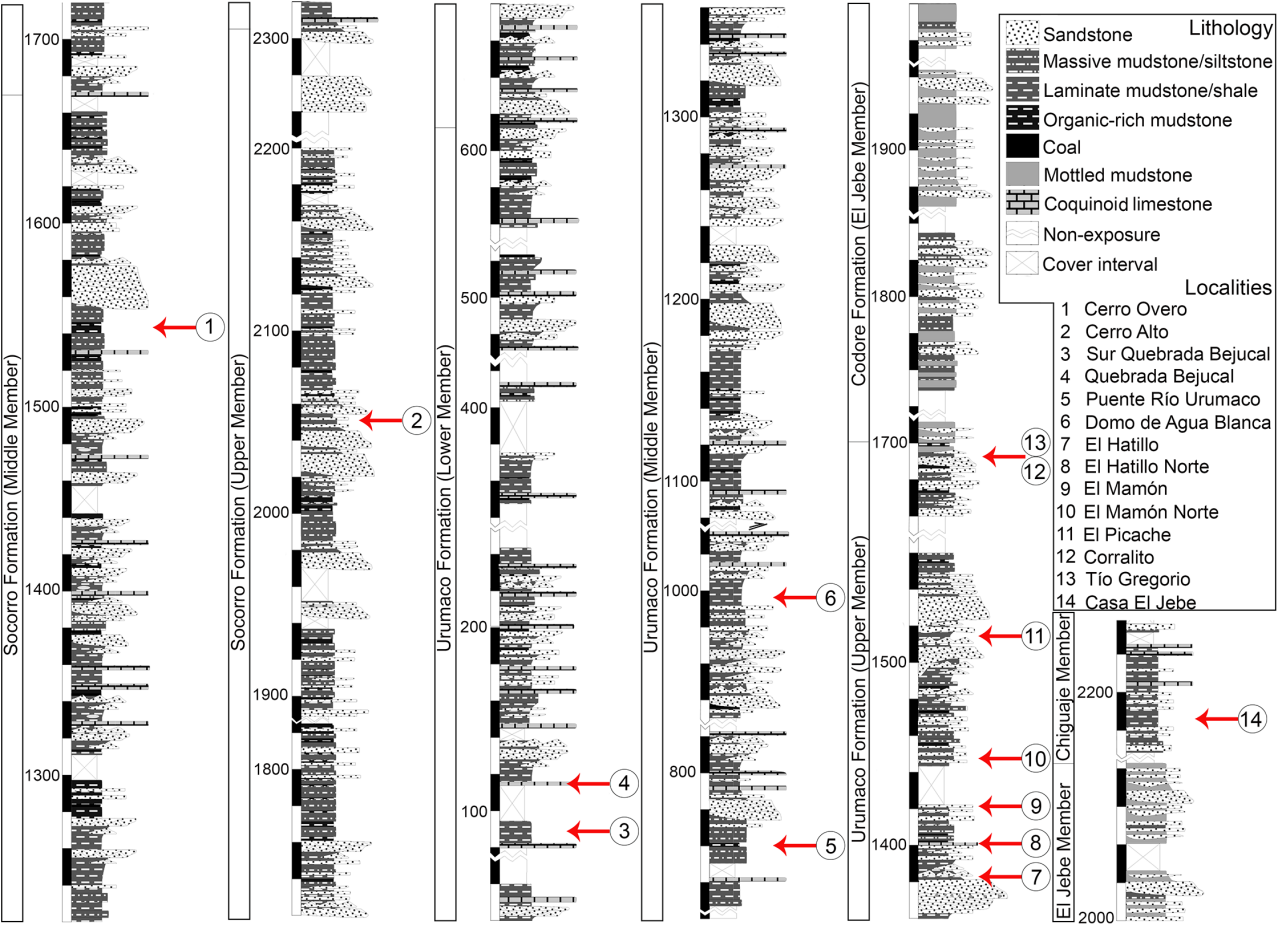
Stratigraphic section of the Socorro, Urumaco and Codore formations. Modified from Quiroz and Jaramillo [27].

The lower and upper contacts of the Socorro Formation (Fig 2) are conformable with the Querales and Urumaco formations, respectively [27, 50, 51]. Hambalek et al. [51] informally divided the Socorro Formation into three members. Later, Quiroz and Jaramillo [27] conducted a detailed sedimentological stratigraphic study, identifying a thickness of 2300 m to the whole Socorro Formation along Paují Creek, 20 km east of the town of Urumaco. The Middle Member is 880 m thick, and is characterized by complex interbedding of medium to fined-grained sandstone, organic mudstone, coal, shales and coquinoidal limestones with abundant mollusks remains [27]. The Upper Member is 639 m thick and it is characterized by gray, massive-bedded, sandy mudstone interbedding with organic mudstones with plants and coal fragments and scarce coquinoidal limestones [27, 51].

### Urumaco Formation (middle–late Miocene)

The lower and upper contacts of the Urumaco Formation (Fig 2) are conformable with the Socorro and Codore formations, respectively [27, 51]. The unit has a thickness of approximately 1700–2060 m [27, 50], and toward the east it is correlative with the Caujarao Formation that crops out in the Coro-La Vela region [27, 50]. The unit has been divided in three members: the Lower Member, 780 m thick, is dominated by dark-gray laminated mudstones and shales with interbedding fine-grained sandstone and some coquinoidal limestone layers; the Middle Member is characterized by interbedding of medium-to fine-grained sandstone, organic-rich mudstone, shales and coquinoidal limestones; the Upper Member is composed of laminated organic-rich mudstone and shales intercalated with very fine grained sandstones [27, 50].

### Codore Formation (late Miocene–Pliocene)

The lower and upper contacts of the Codore Formation (Fig 2) are conformable with the Upper Member of the Urumaco Formation and the basal conglomeratic level of the San Gregorio formations, respectively [27, 52]. The Codore Formation has been divided into three formal members: El Jebe Member with continental deposits 475 m thick, the Chiguaje Member with marine deposits 65 m thick, and the Algodones Member with continental deposits 320 m thick [27, 52, 53]. The marine Chiguaje Member is characterized by dark-gray laminated mudstones interbedding with fine-grained muddy sandstones and coquinoidal limestones with oysters [27]. Rey [53] assigned an early-late Pliocene age to the Chiguaje Member, based on planktonic foraminifera. Hambalek et al. [51] suggested a late Miocene*–*Pliocene age for the same member based on palynomorphs, and recently Smith et al. [54] suggested a late Miocene-middle Pliocene age, based on planktonic foraminifera.

## Results

### Elasmobranch—systematics

The elasmobranch assemblages from the section of the Urumaco sequence studied here include at least 21 taxa attributed to 13 genera, 9 families and 4 orders (S1 Table).

Neoselachii Compagno, 1977 [55]

Galeomorphii Compagno, 1973 [56]

Lamniformes Berg, 1937 [57]

†Otodontidae Glikman, 1964 [58]

†*Carcharocles* Jordan and Hannibal, 1923 [40]

**Fig 3:**
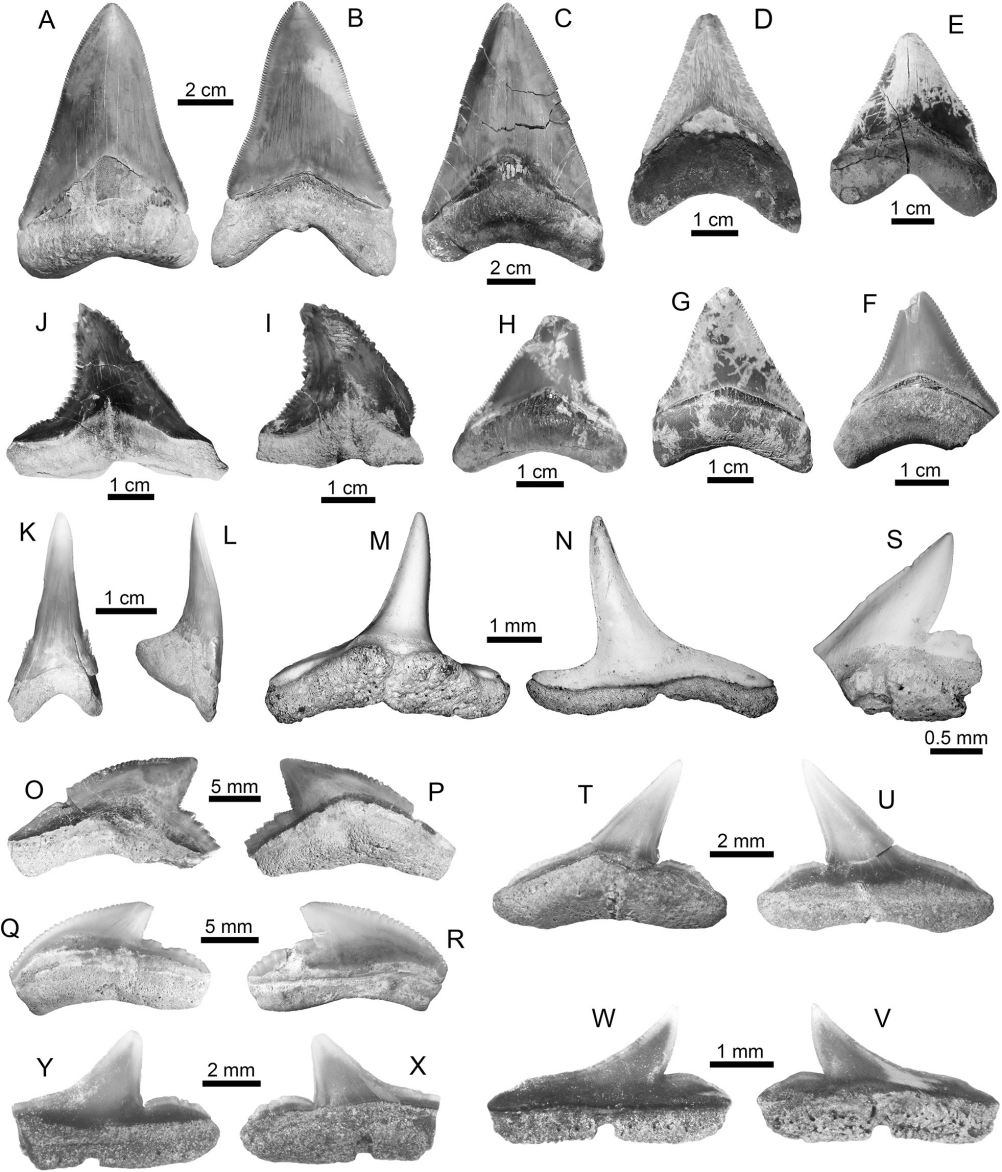
Lamniform and carcharhiniform sharks of the Urumaco sequence. (**A-H**)

†*Carcharocles megalodon* (Agassiz, 1843) [59] (Fig 3A–3H)

#### Description

Fifteen isolated teeth (five upper, seven lower and three of indeterminate jaw position) from the Urumaco (middle–late Miocene) and Codore (late Miocene–Pliocene) formations (S2 Appendix). The crown is triangular in shape, with a flat and convex labial and lingual face, respectively; the lingual face has a typical large neck (inverse “V”-shaped) between the crown and the root. Cutting edges are finely serrated. In all teeth, the root is high, its root lobes are well developed and a weak lingual protuberance is present. The teeth range in height from 23 to 99 mm and width from 35 to 69 mm.

†*Carcharocles megalodon*: Urumaco Fm. [A-B: UNEFM-CIAAP-1292, C: AMU-CURS-455, E: UNEFM-PF-388, F-G: AMU-CURS-605, and H: AMU-CURS-338] and Codore Fm. [D: UNEFM-PF-351)]. (**I-L**) †*Hemipristis serra*: Urumaco Fm. [I: AMU-CURS-332, and J: AMU-CURS-331] and Codore Fm. [K-L: AMU-CURS-331]. (**M-N**) *Paragaleus* sp.: Urumaco Fm. [AMU-CURS-640]. (**O-R**) *Galeocerdo cuvier*: Urumaco Fm. [O-P: UNEFM-PF-408] and Codore Fm. [Q-R: AMU-CURS-625]. (**S-Y**) *Rhizoprionodon* sp.: Urumaco Fm. [T-U: AMU-CURS-497, V-W: AMU-CURS-478, and X-Y: AMU-CURS-408] and Codore Fm. [AMU-CURS-635]. View: labial (B, K, N-O, R, U-W, Y), lingual (A-J, M, P-Q, S-T, V, X), profile (L). Abbreviations: Fm., Formation.

Carcharhiniformes Compagno, 1973 [56]

Hemigaleidae Hasse, 1879 [60]

*Hemipristis* Agassiz, 1835 [59]

†*Hemipristis serra* (Agassiz, 1835) [59] (Fig 3J–3L)

#### Description

Nineteen isolated teeth (nine upper, eight lower and two of indeterminate jaw position) from the Urumaco (middle–late Miocene) and Codore formations (late Miocene–Pliocene) (S2 Appendix). Upper teeth are labio-lingually compressed, with a triangular crown curved distally. The mesial cutting edge is strongly convex and bears fine serrations that end shortly before reaching the apex; distal edge is concave and coarsely serrated with the serrated part also terminating before reaching the apex. The root is high and compressed, with a strong lingual protuberance. Lower teeth with a long and lingually inclined unserrated crown; there are small cusplets near the crown base and the root is bilobate with a strong lingual protuberance. The teeth range in height from 10 to 47 mm and width from 9 to 46 mm.

*Paragaleus* Budker, 1935 [61]

*Paragaleus* sp. (Fig 3M and 3N)

#### Description

One lower antero-lateral tooth from the Urumaco Formation (middle–late Miocene) (S2 Appendix). The crown is prominent and straight, with smooth concave and convex mesial and distal edges, respectively. The distal heel is shorter than the mesial one, and both are low, smooth, without cusps and slightly recurved to the lingual face. The root is low with a flat base, and the lingual protuberance has a well-defined groove. The tooth height is 4 mm and the width is 4.9 mm.

#### Remarks

Four extant *Paragaleus* species are known from the Eastern Atlantic and Indo-Pacific regions [44], and two extinct species have been described to the Miocene of Europe [2]. Extant dentitions of *Paragaleus* spp. show strong dignathic and gradient heterodonty; for this reason a single tooth is unlikely to be diagnostic for species. The tooth AMU-CURS-640 resembles the specimen referred to *Paragaleus* sp. by Carrillo-Briceño et al. [62] from the early Pleistocene of Ecuador.

Carcharhinidae Jordan and Evermann, 1896 [63]

*Galeocerdo* Müller and Henle, 1837 [64]

*Galeocerdo cuvier* (Perón and Lesueur, 1822) [65] (Fig 3O–3R)

#### Description

Four isolated teeth with an unknown jaw position, from the Urumaco (middle–late Miocene) and Codore (late Miocene–Pliocene) formations (S2 Appendix). The crown is triangular and curved distally, the labial face is flat and the lingual one is more convex. The mesial cutting edge is convex and slightly sigmoidal with minor serrations; distal cutting edge is shorter and slightly straight with small serrations. The distal heel is long and strongly serrated. The root is low and slightly curved with a weak lingual protuberance. Complete teeth the range in height from 9 to 13 mm, and in width from 15 to 26 mm.

*Rhizoprionodon* Whitley, 1929 [66]

*Rhizoprionodon* sp. (Fig 3T–3Y)

#### Description

Fourteen isolated teeth (four upper, six lower and four of indeterminate jaw position) from the Urumaco (middle–late Miocene) and Codore (late Miocene–Pliocene) formations (S2 Appendix); most of them are broken and eroded. In upper teeth, the crown is triangular and asymmetric, with a high cusp inclined distally. The mesial cutting edge is slightly concave and the distal cutting edge is slightly straight or sigmoidal; both cutting edges are smooth. The distal heel is high, clearly separated from the cusp by a notch, and this is rounded or finely serrated, depending of the specimen. The root is low with a flat basal face; the labial face is rectilinear and the lingual face has a slight lingual protuberance with a deep medial groove. Complete teeth range in height from 2.5 to 4.4 mm, and in width from 3.5 to 6.3 mm. Due to few diagnostic characters and fragmentary preservation, specimens are not diagnostic to the species level.

*Carcharhinus* Blainville, 1816 [67]

**Fig 4:**
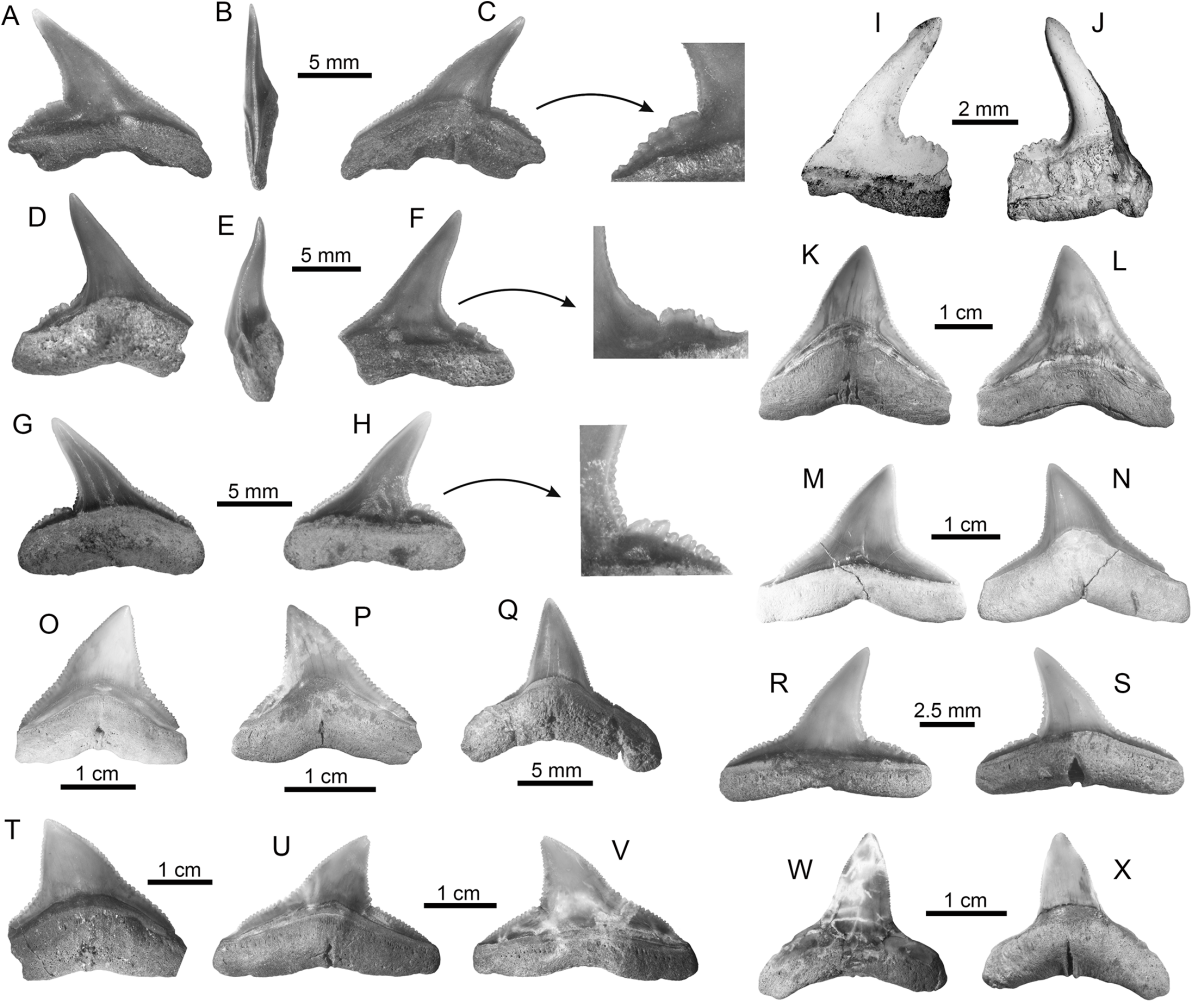
Carcharhiniform sharks of the Urumaco sequence. (**A-J**) †*Carcharhinus caquetius* sp. nov. Holotype: A-C [AMU-CURS-499], paratypes: D-F, I-J [AMU-CURS-477]. (**G-H**) specimen of †*Carcharhinus caquetius* sp. nov. [3406-T-2, 3457-T-(1)] from the Angostura Formation, Ecuador. (**K-Q**) *Carcharhinus leucas*: Urumaco Fm. [K-L: AMU-CURS-360, M-N, Q: AMU-CURS-368], and Codore Fm. [O-P: AMU-CURS-622]. (**R-S**) *Carcharhinus limbatus*: Urumaco Fm. [AMU-CURS-456]. (**T-X**) *Carcharhinus obscurus*: Urumaco Fm. [T: AMU-CURS-464, U-V: AMU-CURS-463, W-X: AMU-CURS-466]. View: labial (A, F, H-I, L-M, R, V-W), lingual (C, D, G, J, K, N-Q, S-U, X), profile (B, E). Abbreviations: Fm., Formation.

†*Carcharhinus caquetius* sp. nov. (Fig 4A–4J)

#### ZooBank life science identifer (LSID) for species

urn:lsid:zoobank.org:act:BE13DF0A-E6F5-44D5-830A-531EE792183D

#### Etymology

The species is named in honor of the Caquetío pre-hispanic tribe who lived in the Falcon state and other parts of western Venezuela.

#### Type locality

Puente Río Urumaco, Urumaco Formation (middle–late Miocene), Middle Member, on the right bank of the Urumaco River (near the Urumaco River bridge), northwestern Venezuela (Figs 1 and 2).

#### Diagnosis

Carcharhinid shark teeth that differ from all other living and extinct *Carcharhinus* species by the following unique combination of dental characteristics: 1) crown with a triangular, strongly elongated and sigmoidal cusp; 2) cusp serrated approximately to its mid-point, with a smooth apex; 3) convex distal heel clearly separated by a deep notch; 4) heel characterized by irregular, small cusps.

#### Referred material

Holotype: upper lateral tooth (AMU-CURS-499) (Fig 4A–4C); Paratypes: two upper lateral teeth (AMU-CURS-477) (Fig 4D–4F and 4I and 4J), all specimens come from the Middle Member of the Urumaco Formation (Puente Río Urumaco locality).

#### Description

The teeth range in height from 6 to7 mm and width from 6 to 7.2 mm, including one upper tooth (3406-T-2) from the Angostura Formation (late Miocene), Ecuador (Fig 4G–4H). Teeth are characterized by a triangular shape with an elongate-sigmoidal, narrow and distally deflected cusp. The lingual face is convex and the labial face slightly convex. The mesial cutting edge is slightly convex and sigmoid, without a clear differentiation between it and the mesial heel; the distal edge is concave in the lower part and sigmoid in the upper part. The mesial crown edge has a strong serration that decreases as it approaches the cusp, giving way to a smooth edge at the apex on both edges of the cusp. The distal heel consists of irregular, small cusps, and is well-separated from the distal edge by a deep notch. The root is thick and slightly concave in the base with slightly rounded lobes; the labial surface is flat and the lingual surface has a shallow lingual protuberance with a shallow lingual groove. To date, we have not found any lower tooth with diagnostic features that justify referral to this new taxon.

#### Remarks

Some extant species of *Carcharhinus*, such as *C*. *borneensis* (Bleeker, 1859) [68], *C*. *dussumieri* (Valenciennes, 1839)[69], *C*. *fitzroyensis* (Whitley, 1943) [70], *C*. *hemiodon* (Valenciennes, 1839) [69], *C*. *porosus* (Ranzani, 1839) [71], *C*. *sealei* (Pietschmann, 1913) [72], *C*. *signatus* (Poey, 1868) [73], and *C*. *sorrah* (Valenciennes, 1839) [69], have upper teeth with a generalized morphological pattern characterized by triangular and distally inclined crowns with coarse serration of the distal heel (e.g., [46]). However, diagnostic elements in the morphology of the cups and the regular serration of the distal heel in the extant taxa previously mentioned permit a clear differentiation between these and the upper teeth of *Carcharhinus caquetius* sp. nov., the teeth of which have a diagnostic elongated, sigmoidal cusp, with a characteristic distal heel conformed by irregular cusps. The extant species most closely related to *C*. *caquetius* sp. nov. could be *C*. *signatus*; nevertheless, in the upper teeth of this taxon, such characters as a strong serration in the mesial heel, a strong and regular serration in the distal heel, a flattened labial face, and a smooth or slightly serrated, wider, shorter and straighter cusp, allow differentiation from *C*. *caquetius* sp. nov. The oldest record of *C*. *signatus* is from the late Miocene of Panama, where a few specimens have been collected from the Chagres Formation [24]. Some species of *Carcharhinus* have ontogenetic and sexual dental variation [2, 43, 46], which makes the taxonomic determination of isolated teeth difficult, especially when only few specimens are available. However, the teeth described as *C*. *caquetius* sp. nov. from the Urumaco Formation, and the specimens from the Angostura Formation (Ecuador), are clearly different from the teeth of other *Carcharhinus* species.

*Carcharhinus leucas* (Müller and Henle, 1839) [ 74 ] (Fig 4K–4Q)

#### Description

Twenty-eight isolated teeth (twenty upper and eight lower) from the Urumaco (middle–late Miocene) and Codore (late Miocene–Pliocene) formations (S2 Appendix). Upper teeth are characterized by crowns with a triangular shape; the lingual face is convex and the labial one is flat. Cutting edges are regularly serrated; the serrations become progressively coarser near the base of the crown. The mesial cutting edge is straight or slightly convex, and the distal one is slightly concave or straight; the distal heel has a typical serrated blade shape. The root is thick and concave along the basal margin. The labial face of the root is flat and the lingual face is convex and is characterized by a weak lingual groove. Lower teeth have a thick and finely serrated crown with the lateral heel finely serrated; the base of the root is slightly concave. The teeth range in height from 12 to 21 mm and width from 10 to 21 mm.

*Carcharhinus limbatus* (Müller and Henle, 1839) [ 74 ] (Fig 4R and 4S)

#### Description

Two upper lateral isolated teeth from the Urumaco Formation (middle–late Miocene) (S2 Appendix). The crown is high, triangular and slightly convex with the apex inclined distally. The lingual face is convex and the labial one is flat. The mesial cutting edge is slightly convex and the distal cutting edge concave; both are serrated. The serrations on the mesial and distal heel are more developed and well-differentiated than the cutting edges. The root is wider that the crown and its base slightly concave; the labial face is flat and the lingual protuberance is slightly developed and has a shallow groove. The teeth range in height from 7 to 12 mm and width from 8 to 10 mm.

*Carcharhinus obscurus* (Lesueur, 1818) [ 75 ] (Fig 4T–4X)

#### Description

Twenty isolated teeth (sixteen upper and four lower) from the Urumaco Formation (middle–late Miocene) (S2 Appendix). Upper teeth are characterized by a triangular shape and lack of an elongate crown with regular serration. The mesial cutting edge is apically convex, with the apex deflected distally; the distal cutting edge is concave. The root is thick, low and slightly concave in the basal margin with slightly rounded lobes; the lingual surface of the root is convex with a shallow lingual groove. Lower teeth have erect crowns finely serrated and moderately arched root lobes. The teeth range in height from 12 to 21 mm and width from 10 to 21 mm.

**Fig 5:**
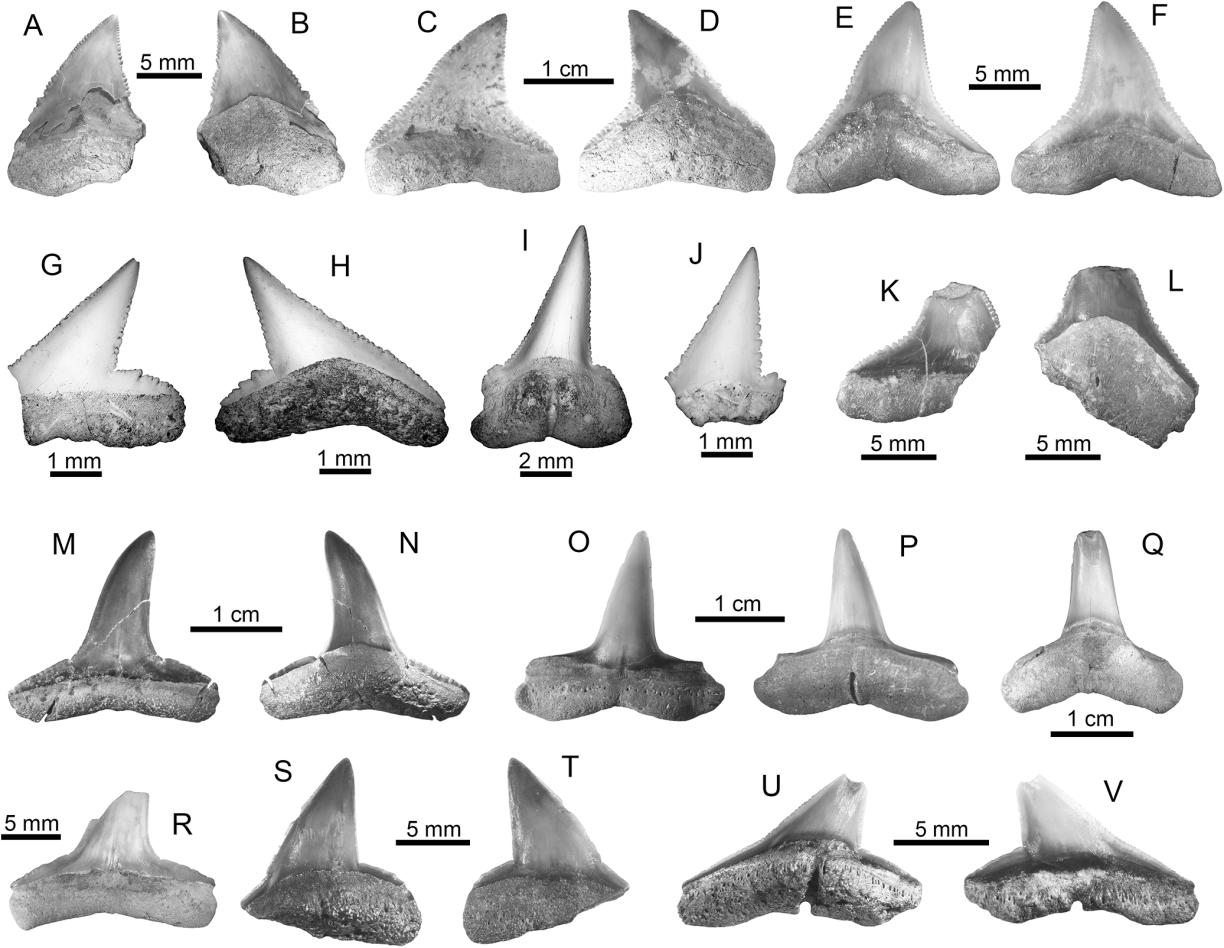
Carcharhiniform sharks of the Urumaco sequence. (**A-F**) *Carcharhinus plumbeus*: Codore Fm. [A-F: AMU-CURS-624]. (**G-J**) *Carcharhinus porosus*: Urumaco Fm. [G-H: AMU-CURS-472, and I: AMU-CURS-590], and Codore Fm. [J: AMU-CURS-631]. (**K-L**) *Carcharhinus* spp.: Socorro Fm. [K-L: AMU-CURS-630]. (**M-R**) *Negaprion brevirostris*: Urumaco Fm. [M-N: AMU-CURS-468, O-P: AMU-CURS-469], and Codore Fm. [Q-R: AMU-CURS-626]. (**S-V**) *Sphyrna* cf. *zygaena*: Urumaco Fm. [AMU-CURS-474; AMU-CURS-477]. View: labial (A, C, F, G, K, M, O, R, T, V), lingual (B, D-E, H-J, L, N, P-Q, S, V). Abbreviations: Fm., Formation.

*Carcharhinus plumbeus* (Nardo, 1827) [ 76 ] (Fig 5A–5F)

#### Description

Four upper isolated teeth from the Codore Formation (late Miocene–Pliocene) (S2 Appendix). The crown is triangular in shape and strongly elongated with the apex deflected slightly distally; the labial face is flat and the lingual one is convex. The convex mesial crown edge is continuous with the mesial heel. The distal edge is convex or slightly straight and can be differentiated from the distal heel, but a notch is lacking. The cutting edges and heels are uniformly and finely serrated. The root base is slightly concave; the lingual face is convex and it is characterized by a lingual groove. The teeth range in height from 15.5 to 18 mm and width from 7 to 22 mm.

*Carcharhinus porosus* (Ranzani, 1839) [71] (Fig 5G–5J)

#### Description

Eight isolated teeth (five upper and three lower) from the Urumaco (middle–late Miocene) and Codore (late Miocene–Pliocene) formations (S2 Appendix). In the upper teeth, the crown is triangular and asymmetric, with a high cusp inclined distally. The mesial edge is straight or lightly concave, and in most of these there is no differentiation between this and the mesial heel; the distal edge is straight. Both cutting edges are strongly serrated, and the serrations decrease in size from the base to the apex. The distal heel is long, strongly serrated and clearly separated from the cusp by a deep notch. The root is low with a slightly concave base. Lower teeth are incomplete, and show oblique, narrow, serrated cusps with well-differentiated and serrated heels. The teeth range in height from 4.5 to 5 mm and width from 5 to 5.2 mm.

*Carcharhinus* spp. (Fig 5K and 5L)

#### Description

Eighty-two incomplete, eroded and non-diagnostic teeth (thirty upper and fifty-two lower) from the Socorro (middle Miocene) and Urumaco (middle–late Miocene) formations (S2 Appendix).

*Negaprion brevirostris* **(**Poey, 1868) [73] (Fig 5M–5R)

#### Description

Twenty-five isolated teeth (fifteen upper and ten lower) from the Socorro (middle Miocene), Urumaco (middle–late Miocene) and Codore (late Miocene–Pliocene) formations (S2 Appendix). Upper teeth show a high and triangular cusp slightly inclined distally. The cutting edges are completely smooth, except the low and weakly serrated lateral heels. The root branches are extended and the basal face is rather broad and flat, with a clear medial lingual groove. Lower teeth are narrower than the upper ones and their heels are generally not serrated. The teeth range in height from 12 to 23 mm and in width from 16 to 22 mm.

Sphyrnidae Gill, 1872 [77]

*Sphyrna* Rafinesque, 1810 [78]

*Sphyrna* cf. *zygaena* (Linnaeus, 1758) [79] (Fig 5S–5V)

#### Description

One upper lateral tooth and one lower lateral tooth from the Urumaco Formation (middle–late Miocene) (S2 Appendix). The upper lateral tooth (height: 12.3 mm) has a high, triangular, asymmetric, broad and distally inclined crown (Fig 5S and 5T); the lingual face is strongly convex and the labial one is flat. The mesial cutting edge is slightly convex and slightly sigmoid and the distal cutting edge is straight; both cutting edges are completely smooth. The distal heel is low and well separated from the cusp by a shallow notch. The root is low with the basal surface slight concave, and is missing the mesial lobe; the lingual protuberance is well developed, bearing a groove. The lower lateral tooth has a stout triangular, slightly serrated crown (Fig 5U and 5V). The lingual face is convex and the labial face is flat. The cusp is inclined distally, being broken and incomplete in the apex. The mesial cutting edge is straight and there is not a clear differentiation between this and the mesial heel; the distal cutting edge is straight. The distal heel is large without serrations and is well separated from the cusp by a shallow notch. The root has a slightly concave base and the lingual protuberance is well developed, bearing a deep groove.

#### Remarks

Juveniles and young adults of extant *Sphyrna zygaena* have teeth with smooth cutting edges, but larger individuals may have slightly serrated teeth [80, 81].

Batomorphii Cappetta, 1980 [82]

Myliobatiformes Compagno, 1973 [56]

**Fig 6:**
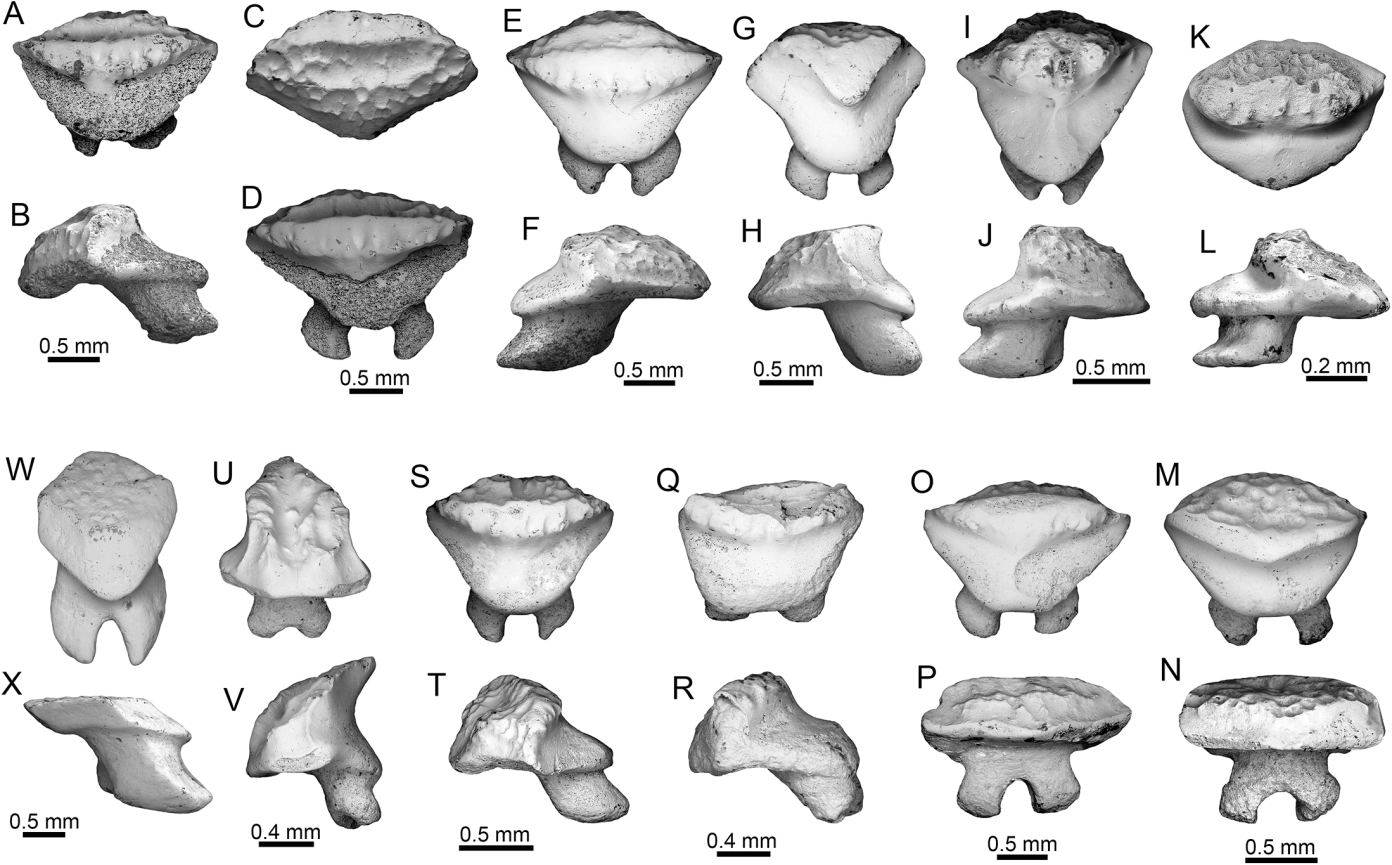
Rays of the Urumaco sequence. (**A-V**) cf. *Dasyatis*: Urumaco Fm. [A-D: AMU-CURS-589, E-F, I-J: AMU-CURS-495, G-H: 496], and Codore Fm. [M-V: AMU-CURS-636]. (**W-X**) Dasyatidae indet.: Urumaco Fm. [AMU-CURS-591]. View: labial (N, P, U), profile (B, F, H, J, L, R, T, V, X), lingual (W), occlusal (A, C-D, E, G, I, K, M, O, Q, S). Abbreviations: Fm., Formation.

Dasyatidae Jordan, 1888 [83]

cf. *Dasyatis* Rafinesque, 1810 [78] (Fig 6A–6V)

#### Description

Sixteen isolated teeth of indeterminate jaw position from the Urumaco (middle–late Miocene) and Codore (late Miocene–Pliocene) formations (S2 Appendix). The teeth are small, only a few millimeters wide. The crown shows a middle transverse crest that separates the labial and lingual faces; the crest is lingually elongate and forms a distinctive cusp in male teeth (e.g. Fig 6G–6J, 6U and 6V). The labial face is sub-triangular in shape, with a distinctive alveolate ornamentation, and a labio-lingually extended depression; the lingual face is smooth and is divided into two lingual marginal areas by a lingual ridge. The root has two lobes, which are lingually arched with a flat or slightly bulging base. There is a foramen in the middle of the sulcus.

#### Remarks

*Dasyatis* shows a dental morphological diversity with distinct gynandric heterodont patterns in most species, which may be more complex at the generic level than currently accepted [2]. Knowledge of dental patterns in extant and fossil *Dasyatis* and others Dasyatidae is still scarce, making any taxonomic assignment of fossil specimens to species difficult.

Dasyatidae indet. (Fig 6W–6X)

#### Description

One small tooth (less than 2 mm length) with a rhomboid and labial-lingually elongated crown from the Urumaco Formation (middle–late Miocene). The occlusal surface is flat and appears to be eroded; however, this has a light alveolate ornamentation. The root is massive and presents two distinct lobes with a flat basal faces that becomes narrower lingually; between these there is a deep sulcus with two foramina. Due similarities in dental morphology among dasyatid genera and the few fossil specimens referred herein, we maintain this specimen in open nomenclature.

Myliobatidae Bonaparte, 1838 [84]

*Aetobatus* Blainville, 1816 [67]

**Fig 7:**
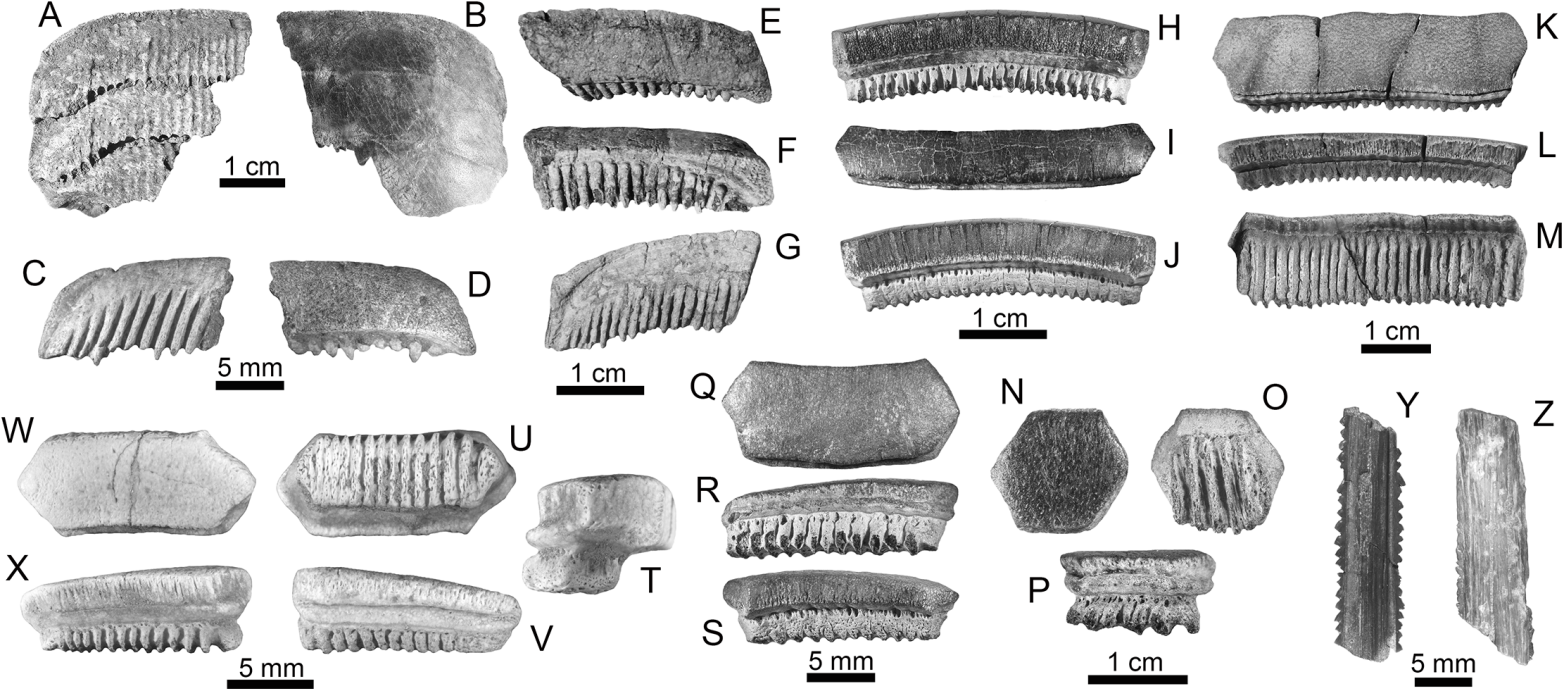
Rays of the Urumaco sequence. (**A-G**) *Aetobatus* cf. *narinari*: Urumaco Fm. [E-G: AMU-CURS-308] and Codore Fm. [A-B: AMU-CURS-598, and C-D: AMU-CURS-614]. (**H-J**) *Myliobatis* sp.: Urumaco Fm. [AMU-CURS-487]. (**K-X**) *Rhinoptera* sp.: Urumaco Fm. [K-P: AMU-CURS-288, and Q-S: AMU-CURS-489], and Codore Fm. [T-X: AMU-CURS-621]. (**Y-Z**) Myliobatiformes indet.: Urumaco Fm. [Y: AMU-CURS-492], and Codore Fm. [Z: AMU-CURS-634]. View: basal (A, C, G, M, O, U), dorsal (Z), labial (J, L, S, X), profile (T), lingual (F, H, P, R, V), occlusal (B, D-E, I, K, N, Q,), oblique occlusal (W), ventral (Y). Abbreviations: Fm., Formation.

*Aetobatus* cf. *narinari* (Euphrasen, 1790) [85] (Fig 7A–7G)

#### Description

Fifty-seven incomplete, isolated teeth (eight upper and forty-nine lower) and two fragmented upper dental plates, from the Socorro (middle Miocene), Urumaco (middle–late Miocene) and Codore (late Miocene–Pliocene) formations (S2 Appendix). Upper teeth are almost straight and the lower ones are mesio-distally V-shaped. The crown is labio-lingually thicker in the central than in the lateral region. In occlusal view, the crown surface is smooth and the labial and lingual margins are vertical and strongly ornamented; on the lingual margin there are well marked alternating furrows and laminae, whereas these structures are not so evident on the labial one. The root is polyaulacorhize, apico-basally flattened and decreases in height to the lateral edges of the tooth, and is displaced lingually with respect to the crown.

#### Remarks

Though the teeth from the Urumaco sequence described here are not adequately preserved, their tooth morphology has strong resemblance to those of the recent species *Aetobatus narinari* [86]. At least two nominal species of *Aetobatus* were described from early to late Miocene deposits of Europe: *Aetobatus arcuatus* (Agassiz, 1843) [59] and *Aetobatus cappettai* Antunes & Balbino, 2006 [87]. *Aetobatus arcuatus* is possibly restricted to the early-middle Miocene, and *A*. *cappettai* has only been reported from the late Miocene of Portugal [88]. For a recent discussion of Miocene *Aetobatus* teeth, see Bor et al. [89].

*Myliobatis* Cuvier, 1816 [90]

*Myliobatis* sp. (Fig 7H–7J)

#### Description

With 798 teeth (indeterminate upper-lower jaw position), this is the most abundant taxon sampled from the Urumaco (middle–late Miocene) and Codore (late Miocene–Pliocene) formations (S2 Appendix). The specimens consist of complete and fragmented isolated teeth, mainly from the medial (780 specimens) and lateral tooth row (18 specimens). The medial teeth are broader (mesio-distally) than long (labio-lingually), with a hexagonal contour, and in most cases are rectilinear, but some are arched. Teeth of lateral and posterior files are longer than broad, with a hexagonal, pentagonal or triangular contour depending on their position in the files. In all teeth, the occlusal crown face is practically flat and the labial and lingual margins are slightly ornamented; the root has a polyaulacorhize vascularization type. Complete teeth range in width from 8 to 57 mm, and height from 3 to 10 mm.

#### Remarks

Taxonomic identification based on isolated teeth of *Myliobatis* is difficult due to the large dental variation within the group [86, 91].

Rhinopteridae Jordan and Evermann, 1896 [63]

*Rhinoptera* Cuvier, 1829 [92]

*Rhinoptera* sp. (Fig 7K–7X)

#### Description

Fifty complete and incomplete isolated teeth (14 mesial and 36 laterals of indeterminate upper-lower jaw position) recovered from the Urumaco (middle–late Miocene) and Codore (late Miocene–Pliocene) formations (S2 Appendix). The crown is variably high, hexagonal, straight or slightly convex labially. The occlusal surface is flat and there is a thick basal ledge on the lingual face of the crown. Lateral teeth are less enlarged than the medial ones, showing a regular hexagonal shape; the distal crown height is typically lower than mesial height. The root has polyaulacorhize vascularization type, with a quadrangular shape, and does not project beyond the level of the crown. Complete teeth range in width from 13 to 38 mm, and height from 3 to 9 mm.

#### Remarks

As in *Myliobatis*, the taxonomic identification of isolated teeth of *Rhinoptera* is extremely difficult due to extensive dental variation in the group (e.g., [86]).

Myliobatiformes indet. (Fig 7Y and 7Z)

#### Description

Six fragmented caudal spines from the Urumaco (middle–late Miocene) and Codore (late Miocene–Pliocene) formations (S2 Appendix). The specimens preserved an evident dorsal groove, a ventral central ridge and strong serrations along both sides of the spine.

#### Remarks

The majority of myliobatiforms have caudal spines and some characters can be used to identify fossil taxa [86]; however due the fragmentary condition of the specimens referred herein, these characters cannot be usually observed.

Rajiformes Berg 1937 [57]

Rhynchobatidae Garman, 1913 [93]

*Rhynchobatus* Müller and Henle 1837 [64]

**Fig 8:**
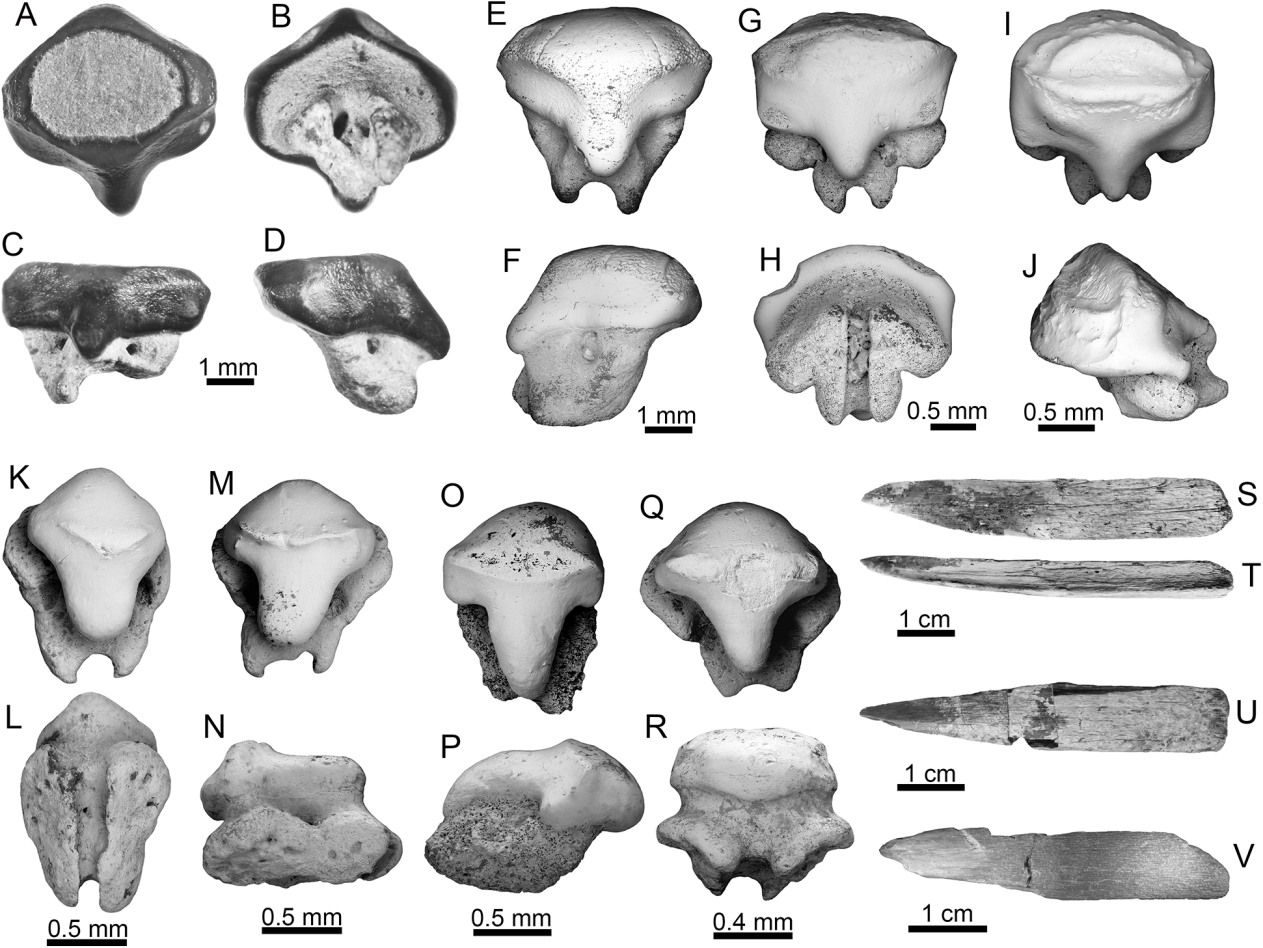
Guitarfish and sawfish of the Urumaco sequence. (**A-J**) *Rhynchobatus* spp.: Urumaco Fm. [A-D: AMU-CURS-482 and E-F: AMU-CURS-628], Codore Fm. [G-J: AMU-CURS-638]. (**K-V**) *Pristis* sp. Buccal teeth: Urumaco Fm. [K-R: AMU-CURS-484], and rostral spines: Urumaco Fm. [S-T: AMU-CURS-243 and U: AMU-CURS-244], and Codore Fm. [V: AMU-CURS-620]. View: basal (B, H, L), dorsal (S), labial (R), profile (D, F, J, N, P), lingual (C,), occlusal (A, I, K, M, O, Q), oblique occlusal (E, G), ventral (U-V), posterior (T). Abbreviations: Fm., Formation.

*Rhynchobatus* spp. (Fig 8A–8J)

#### Description

Three isolated teeth of indeterminate jaw position from the Urumaco Formation (middle–late Miocene), and two from the Codore Formation (late Miocene–Pliocene) (S2 Appendix). Teeth from the Urumaco Formation have a globular and lozenge-shaped crown with rounded edges and with a fine granular ornamentation. The labial face is strongly convex and the lingual face presents an uvula that extends beyond the root sulcus. The uvula is flanked on both sides by a slight depression. The occlusal part of the crown in one of the specimens is flattened due to wear (Fig 8A–8D). The root is massive and presents two distinct lobes with a flat basal face that becomes narrower lingually; between these there is a root sulcus with two foramina. The teeth range in width is from 4.9 to 5 mm, and height from 4.7 to 5 mm. The two specimens from the Codore Formation have a globular and lozenge-shaped crown with rounded edges. The labial face is strongly convex and the lingual face presents an uvula that extends beyond the root sulcus. Both specimens have a very prominent arched transverse keel on the crown. The specimen AMU-CURS-638-a (Fig 8I and 8J) shows ridges and a broad, shallow depression on the labial face of the crown. The root is massive and presents two distinct lobes with flat basal faces that become narrower lingually; between these there is a root sulcus with two foramina. The teeth range in width from 2 to 2.1 mm, and height ranges from 1.9 to 2 mm.

#### Remarks

The specimen AMU-CURS-477-a has morphology similar to an isolated tooth from the late Miocene of Panama referred to *Rhynchobatus* sp. by Pimiento et al. [23]. Because of the range of dental variation in extant species is unknown, and recovered specimens are rare and poor preserved, we refrain from taxonomic identification at species level.

### Taxonomic and morphological features of sawfish material in the sequence

**Fig 9:**
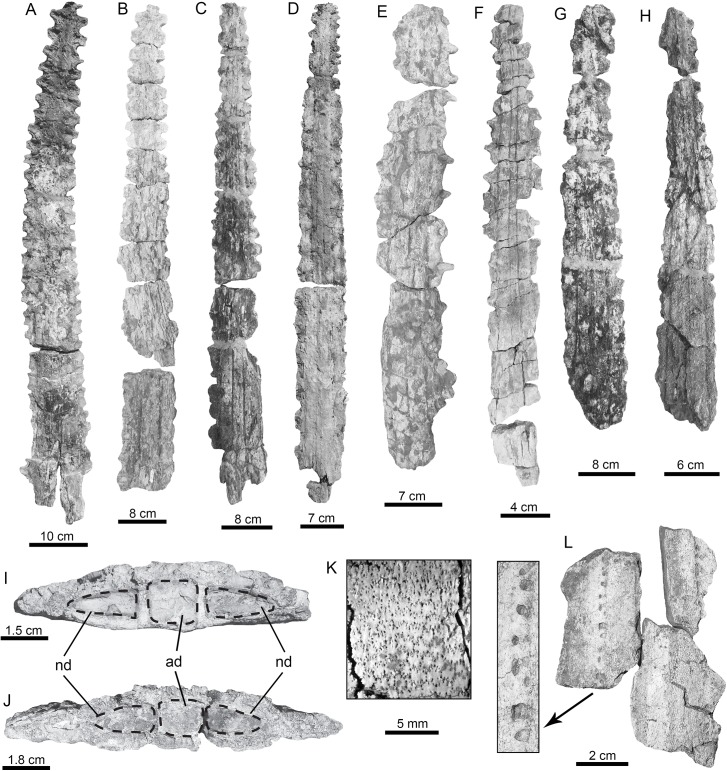
Sawfish rostra of the Urumaco sequence. (**A-L**) *Pristis* sp. rostra: Socorro Fm. [E: AMU-CURS-639 and F: AMU-CURS-241] and Urumaco Fm. [A: AMU-CURS-023, B: AMU-CURS-043, C: AMU-CURS-235, D: AMU-CURS-237, G: AMU-CURS-236, H: AMU-CURS-238 and L: AMU-CURS-251]. (**I-J**) transverse section: [I: AMU-CURS-043 and AMU-CURS-376, Urumaco Fm.]. (**K**) mineralized cartilage: [AMU-CURS-235]. (**L**) fragment of rostrum showing the depressions and foramina interpreted as ampullae of Lorenzini. View: dorsal (A-H), posterior (I-J). Abbreviations: Fm., Formation; ad, axial duct; nd: neural ducts.

The sawfish specimens (Pristidae Bonaparte, 1838 [84]) are represented by two partial rostra from the Socorro Formation (middle Miocene); four buccal teeth, one complete rostrum, 21 partial rostra, 42 rostral spines from de Urumaco Formation (middle–late Miocene), and one rostral spine from the Codore Formation (late Miocene–Pliocene) (S2 Appendix). We examined multiple morphological features, including buccal teeth (Fig 8K–8R), rostral spine shape (Fig 8S–8V), alveolar shape, and the ratio between the total rostral length and the standard rostral length. All specimens recovered from the Miocene of the Urumaco sequence are referable to *Pristis* Linck, 1790 [94] (Figs 8K–8R, 8S–8V and 9A–9L) rather than to *Anoxypristis* White and Moy-Thomas, 1940 [95], based on the internal morphology of the rostrum, as well as the shape of the spines [41]. The most complete specimen from the Urumaco sequence is AMU-CURS-023 (Urumaco Formation, Lower Member) (Fig 9A), previously referred by Aguilera [19] to the extant species *Pristis pectinata* Latham, 1794 [96]. Based on our study of numerous aspects of morphology, the pristids from the Urumaco sequence fall within the rostral morphospace of modern *Pristis* species, however are not easily referable to any one fossil or extant species.

#### Buccal tooth morphology

The four buccal teeth of indeterminate jaw position (Urumaco Formation, Middle Member) (Fig 8K–8R) are less than 1 mm long. The crown is semiglobular and rounded, with a transverse crest and a long uvula that does not extend beyond the root. The root is much broader than the crown; the furrow is broad and has a large foramen. Oral teeth are somewhat difficult to identify because those of extant species are poorly known and are morphologically variable [2]. According to Cappetta [2], teeth of *Pristis pristis* (Linnaeus, 1758) [79], are longer than broad; however, the teeth of *Pristis pectinata* illustrated by Herman et al. [97], show variability in length and shapes. The specimen from the Urumaco Formation also differs from the buccal teeth of *Pristis aquitanicus* Delfortrie, 1872 [98], described from the early-middle Miocene of France, and illustrated by Cappetta [99].

#### Rostral spine morphology

Although often referred to as teeth, the rostral spines are not homologous with the buccal teeth of other vertebrates and represent an independent derivation from highly modified scales [100]. Isolated rostral spines (Fig 8S–8V) have a length up to 95 mm; these are generally long, pointed and slightly curved distally. The anterior edge is sharp and the posterior edge is concave. These rostral spines are rarely preserved in association with rostra from the Urumaco sequence. An exception to this is the specimen AMU-CURS-251 (Urumaco Fm., Upper Member), which preserves a single associated spine, and AMU-CURS-237 and AMU-CURS-639 (Socorro Formation, Upper Member), in which multiple spines are preserved, but are heavily damaged and all lack distal tips. The spines of AMU-CURS-639 are robust, and both the rostral spines of this specimen and the isolated rostral spine of AMU-CURS-376 have a rounded anterior margin and a grooved posterior margin. These features are consistent with *Pristis*, as noted by Cappetta [2] and inconsistent with *Anoxypristis*. Alveoli are anteroposteriorly asymmetrical, with convex anterior edges and weakly concave posterior edges.

#### Rostral spine count

An accurate rostral spine count was available only for the rostrum AMU-CURS-023 (Fig 9A). In this specimen, 24 alveoli were found on the right-hand side of the rostrum, and 23 on the left. This count is notably lower than the count of 26 recorded by Aguilera [19] for the same specimen. Counts of 23 and 24 are consistent with every extant pristid [49, 101] (Table 1) although at the lower limit of the range for *Pristis zijsron* Bleeker, 1851 [102], and the high end of the range of *P*. *pristis*. Rostral spine counts appear to vary on a regional basis in pristids, and the Recent Western Atlantic population of *P*. *pristis* has a lower count than other populations. In contrast, the Western Atlantic population of *P*. *pectinata* has a higher count [103]. The specimen AMU-CURS-043 (Urumaco Formation, no collections data about member) (Fig 9B), although incomplete posteriorly and with erosion of the posterior penultimate segment preventing any count from this certainly spine-bearing region, shows a count of 22 alveoli on the best preserved side. The specimens AMU-CURS-235 and AMU-CURS-237 (both from the Urumaco Fm., Upper Member) (Fig 9C and 9D), although less well-preserved than AMU-CURS-023 and with some uncertainty regarding posterior completeness, have minimum counts of 23 and 20 alveoli on the right side, respectively (best preserved side).

**Table 1:** Measurements and ratios for extant *Pristis* spp. and rostra from the Urumaco sequence.

	Rostral spine count	SRL	SRW (TW)	DS	DPS	SRW/SRL	TW/SRL	SRL/TRL
*Anoxypristis*	16–33			1.1–2.0	0.16–0.44	0.08–0.15	0.05–0.08	0.59–0.81
*P*. *pristis*	14–24			2.0–2.9	0.53–1.05	0.15–0.25	0.06–0.1	0.91–0.98
*P*. *clavata*	18–27			1.2–2.0	0.25–0.7	0.16–0.22	0.07–0.11	0.89–0.97
*P*. *zijsron*	23–37			1.1–2.0	0.1–0.33	0.09–0.17	0.04–0.09	0.85–0.97
*P*. *pectinata*	20–30			2.1	0.21–0.41	0.13–0.15	0.07–0.08	0.86–0.99
*P*. *atlanticus* ^†^	~20	116.3	14.5 (7.0)	—	0.29	0.12	0.06	—
AMU-CURS-023	23/24	70.9	10.7 (5.5)	2.0	0.57	0.15	0.08	0.88
AMU-CURS-235	23	57.7	7.6* (3.4)	—	0.25	0.13	0.06	—
AMU-CURS-237	20+	75.0	8.1 (4.5)	1.65	0.52	0.11	0.06	—

Data from Whitty et al. [49], except *P*. *pectinata* ratios which are from 14 individuals listed in Robillard and Séret [116], and *P*. *atlanticus* (rostral spine estimate from Casier [108]; measurement data from Zbyszewski [117]).

*Estimate based on doubling the width of the preserved half.

DS = sum of the distal and next most distal interalveolar ratios from the right and left sides; DPS score = ratio between the spacing of the most anterior two and most posterior two rostral spines; SRL = standard rostral length; SRW = standard rostral width; TRL = total rostral length; TW = tip width

† = extinct taxon.

#### Internal duct morphology

Several of the *Pristis* rostra from the Urumaco sequence preserve the internal rostral cavities, often filled with sedimentary material that increases contrast with the rostral cartilages (Fig 9I and 9J). Anteriorly, only the ducts for the ophthalmic and buccal nerves (following Wueringer et al. [41]) are present; the precranial cavity is absent. The neural ducts are roughly ovate, tapering slightly laterally. Posterior to this, the precranial cavity first appears as a dorsal and a ventral opening. Preservation does not permit assessment as to whether these are connected by a medial constriction, or separated by mineralized cartilage (AMU-CURS-241 and AMU-CURS-376); however slightly more posteriorly they are joined by a medial constriction, and the dorsal and ventral portions are expanded parallel to the rostral margins (AMU-CURS-376) (Fig 9J). The precranial cavity becomes square in outline, and increases in size. The neural ducts develop flattened medial edges, and more tapered lateral edges. The dorsal surface of the axial duct is embayed to contain a small foramen located dorsal to the precranial cavity. This secondary duct in the precranial cavity is found in all adequately preserved rostra from the Socorro and Urumaco formations, and has also been figured in some extant *Pristis* material (e.g. [104]). Its presence has been explained as an artifact relating to shrinking of the hyaline cartilage during the preparation of dried specimens [105], and a similar explanation cannot be ruled out in the fossil material. The structure of the internal rostral cavities is broadly similar to those described and illustrated for *Pristis* by Duméril [104], Hoffmann [105], Kirkland and Aguillón-Martínez [106], Cicimurri [107], and Wueringer et al. [41]. The presence of a single, oval to tear-drop shaped neural duct as opposed to two circular ducts differentiates the genera *Pristis* and *Anoxypristis* [41].

#### Mineralized cartilage organization

The *Pristis* rostra from Socorro and Urumaco formations share a complex pattern of rostral mineralization. There are two thick layers of prismatic calcified cartilage surrounding the external surface of the rostrum, forming embayments for the alveoli and surrounding the neural canals. On the inner surface of the neural canals, a third layer is present, thinner than the two thus described and not as clearly composed of discrete prisms. There also appears to be an additional, very thin layer of mineralized tissue on the external surface of the rostrum. As many of the rostra are quite weathered, this feature can be seen in only a few specimens. It has a fibrous appearance in macroscopic view. Recent sawfish rostra have been described as consisting of three mineralized cartilage layers [105, 108]: an external prismatic layer and prismatic layers encircling the neural ducts, and a layer lining the neural ducts. Hoffmann [105] mentioned the fibrous outer layer of connective tissue, but did not suggest that it was normally mineralized; however this outer mineralized layer was preserved in *Pristis propinquidens* Casier, 1949 [108]. Many authors failed to note the mineralized layer lining the neural ducts [41, 106], but the studies of Hoffmann [105] and Casier [108], demonstrated that this mineralized layer is present in both Recent and fossil *Pristis* species.

#### Ampullae of Lorenzini

Depressions and foramina are visible in the mineralized fibrous layer on the ventral surface and immediately lateral to the neural duct of one of the more exceptionally preserved rostra AMU-CURS-251 (Fig 9L). These are distributed in approximately two closely spaced rows parallel to the edge of the neural duct, and are interpreted as housing the ampullae of Lorenzini, with the foramina transmitting the sensory nerves from the lateral line and ampullae of Lorenzini. These depressions were interpreted as related to electroreception rather than to the lateral line system, as the latter, while occasionally associated with subdermal tissues [109] is described as being composed of tubules rather than clusters of receptors. In contrast, the ampullary clusters are not part of a continuous tubular system and are described specifically as being embedded in connective tissue [110]. Differences exist in the distribution pattern of the ampullae of Lorenzini between *Anoxypristis*, *Pristis pristis* and *P*. *clavata* Garman, 1906 [111] (e.g. [110]). There is no information available as to whether the distribution of ampullae in the intact rostrum mirrors the pattern of fossae in the fibrous outer mineralized layer of the ventral surface of the rostrum. Assuming that it does, the distribution of ampullae seen in the *Pristis* material from the Socorro and Urumaco formations is closer to that of smalltooth sawfish (*P*. *zijsron*, *P*. *pectinata*, and *P*. *clavata*) than to *P*. *pristis*.

## Discussion

### Taxonomic composition

The elasmobranch faunal assemblages described here from the Urumaco sequence include at least 21 taxa (S1 Table), of which only three are extinct (*Carcharocles megalodon*, *Hemipristis serra* and *Carcharhinus caquetius* sp. nov.). From the complete assemblages, two taxa were found to be new fossil records for Venezuela and the Caribbean region (*Carcharhinus caquetius* sp. nov., and *Carcharhinus porosus*). The remaining taxa have been recorded from other Neogene marine deposits of Venezuela [20], Central, North, and South America [18, 24, 62]. Many of the taxa from the Urumaco sequence have also been found in Neogene rocks around the world [2], confirming the cosmopolitan distribution of many of these species during the Miocene–Pliocene.

The elasmobranch fauna of the Urumaco sequence shows a clear differentiation in paleodiversity between geologic units. The Urumaco Formation, with 11 localities, has a paleodiversity of 20 taxa, while the Codore Formation, with only one studied locality, is characterized by 16 taxa and the Socorro Formation only by six taxa (S1 Table). The low paleodiversity of the Socorro Formation in comparison with the other units could be attributable to less intensive sampling. In spite of their absence from the Urumaco sequence, taxa such as *Carcharocles megalodon* and *H*. *serra* have been found in the Socorro Formation at outcrops located closer to Coro City [3].

Fossil taxa such as *Galeocerdo cuvier*, *Rhizoprionodon* sp, *Carcharhinus leucas*, *C*. *limbatus*, *C*. *obscurus*, *C*. *plumbeus*, *C*. *porosus*, *Negaprion brevirostris*, *Sphyrna* cf. *zygaena*, *Sphyrna* sp., cf. *Dasyatis*, *Aetobatus* cf. *narinari*, *Myliobatis* sp., *Rhinoptera* sp., and *Pristis* sp. have extant counterparts in tropical America and adjacent regions; other taxa such as the genus *Rhynchobatus* live exclusively in the eastern Atlantic (off the African coast) and Indo-West Pacific [112]. The occurrence of *Rhynchobatus* in the marine facies of the Urumaco and Codore formations and in other geologic units of Venezuela [20], Costa Rica [10], and Panamá [23] confirms the presence of this taxon in the Caribbean region during the late Miocene, suggesting that it became extinct from the proto-Caribbean Sea and western Atlantic, possibly as a consequence of environmental changes during the final stage of closure of the Panamanian isthmus [25], or due to competition with other species. The presence of *Carcharhinus caquetius* sp. nov. in the late Miocene of Ecuador suggests a neritic distribution in the proto-Caribbean and Eastern Pacific for this species.

### Taxonomic and morphological features of sawfish

There are four valid extant species of *Pristis*, *P*. *pristis*, *P*. *clavata*, *P*. *zijsron* and *P*. *pectinata*, supported by morphological and molecular data [103]. *P*. *pristis*, with a circumtropical distribution, is the sister taxon to the others (“smalltooth sawfish”), all of which show more localized distributions either in the Atlantic-Caribbean (*P*. *pectinata*) or the Indo-West Pacific (other species). The number and spacing of rostral spines has some discriminatory power in identifying the extant species of *Pristis*, although extensive variation within and between populations has been noted [101, 103]. The number of spine positions is determined early in embryological development [113], however, the environmental and developmental factors underlying the number and position of the rostral spines are unknown.

Whether all *Pristis* material from the Urumaco sequence can be referred to a single species must be considered. This question is especially relevant since modern sawfish species have overlapping geographic ranges [101], and in historic times, the living species *P*. *pristis* and *P*. *pectinata* were present sympatrically in the Caribbean and Gulf of Mexico [114, 115]. At present, there seems to be little evidence for two fossil species in the Urumaco Formation, although the range of DPS values is quite broad (Table 1), especially including the specimen AMU-CURS-235. However, based on other metrics this specimen is generally consistent with other material from the locality, and due to poor preservation on the posterior right side, it is possible that rostral spine spacing is anomalous on the left and is giving a false signal.

Based on our study of numerous aspects of morphology, the Miocene pristids from the Urumaco sequence fall within the rostral morphospace of modern *Pristis* spp., nevertheless, are not easily referable to any one extant species. The *Pristis* material from the Urumaco sequence shows overlap in the number of rostral teeth with *P*. *pristis*, although at the upper end of the range of this species. Nonetheless, if the whole Venezuelan collection is considered as a single species, DS and DPS values lie outside of the recorded range of *P*. *pristis* (Table 1). In addition, the posterior rostrum is more slender (SRW/SRL), and the space between the most posterior rostral spine and the chondrocranium is greater (TRL/SRL). In general, the combination of these variables suggests that the fossil material of sawfish from the Urumaco sequence is inconsistent with *P*. *pristis*, and should be included within the smalltooth sawfish clade.

Within the smalltooth sawfishes, *P*. *clavata* has a more robust rostrum with a shorter space anterior to the chondrocranium than the Urumaco sequence material (Table 1). Generally high DPS scores and lower rostral spine counts also make referral to *P*. *zijsron* unlikely. While geographic distribution is most consistent with *P*. *pectinata*, the high DPS score and slightly narrower rostra of the best-preserved Urumaco material creates uncertainty. *P*. *zijsron* and *P*. *pectinata* are sister taxa [103], and there may have been less differentiation in rostral morphospace occupation in Miocene smalltooth sawfish of this lineage, including the Urumaco rostra and *P*. *atlanticus* from the middle Miocene of Portugal [117].

The total rostral length in *Pristis* is generally considered to be between 1/3 and 1/5 of the total length of the fish [118], and provides a broad size estimate of between 2.4–4 m total length for AMU-CURS-023, 2.1–3.5 m total length for AMU-CURS-235, and 2.7–4.8 m total length (AMU-CURS-237). *P*. *pectinata* is thought to reach maturity at 2.7–3.6 m, and has a maximum length of over 6.0 m [119]. It seems likely that AMU-CURS-023 and AMU-CURS-237 are adults, and AMU-CURS-235 is either a large juvenile or small adult, but that all are within the size range predicted by extant *Pristis* species, and specifically *P*. *pectinata*.

### Paleoenvironment and paleoecology

**Fig 10:**
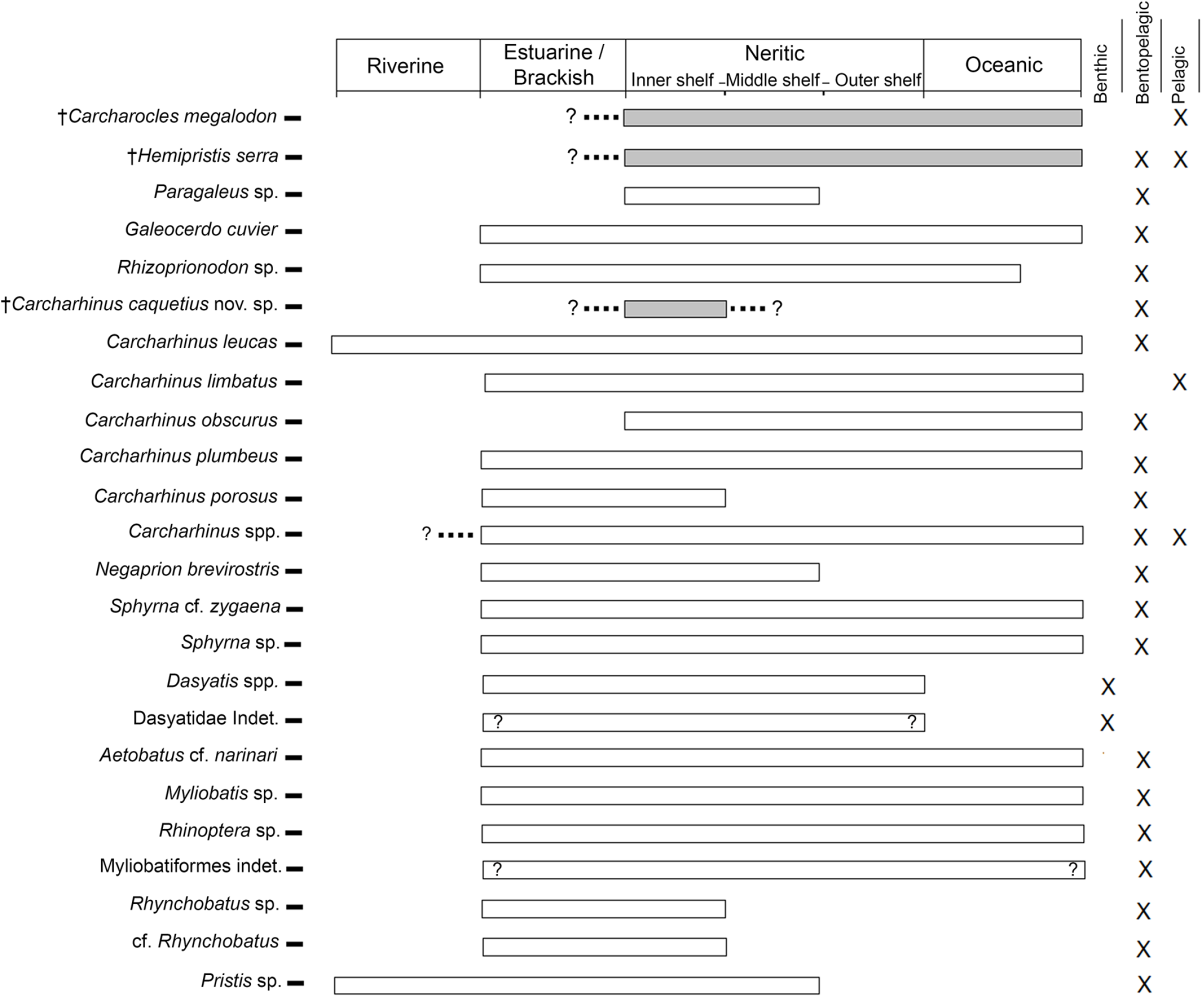
Habitat preferences of Urumaco sequence elasmobranch taxa, based on preferences of extant relatives. Light-gray shading indicates extinct taxa.

**Fig 11:**
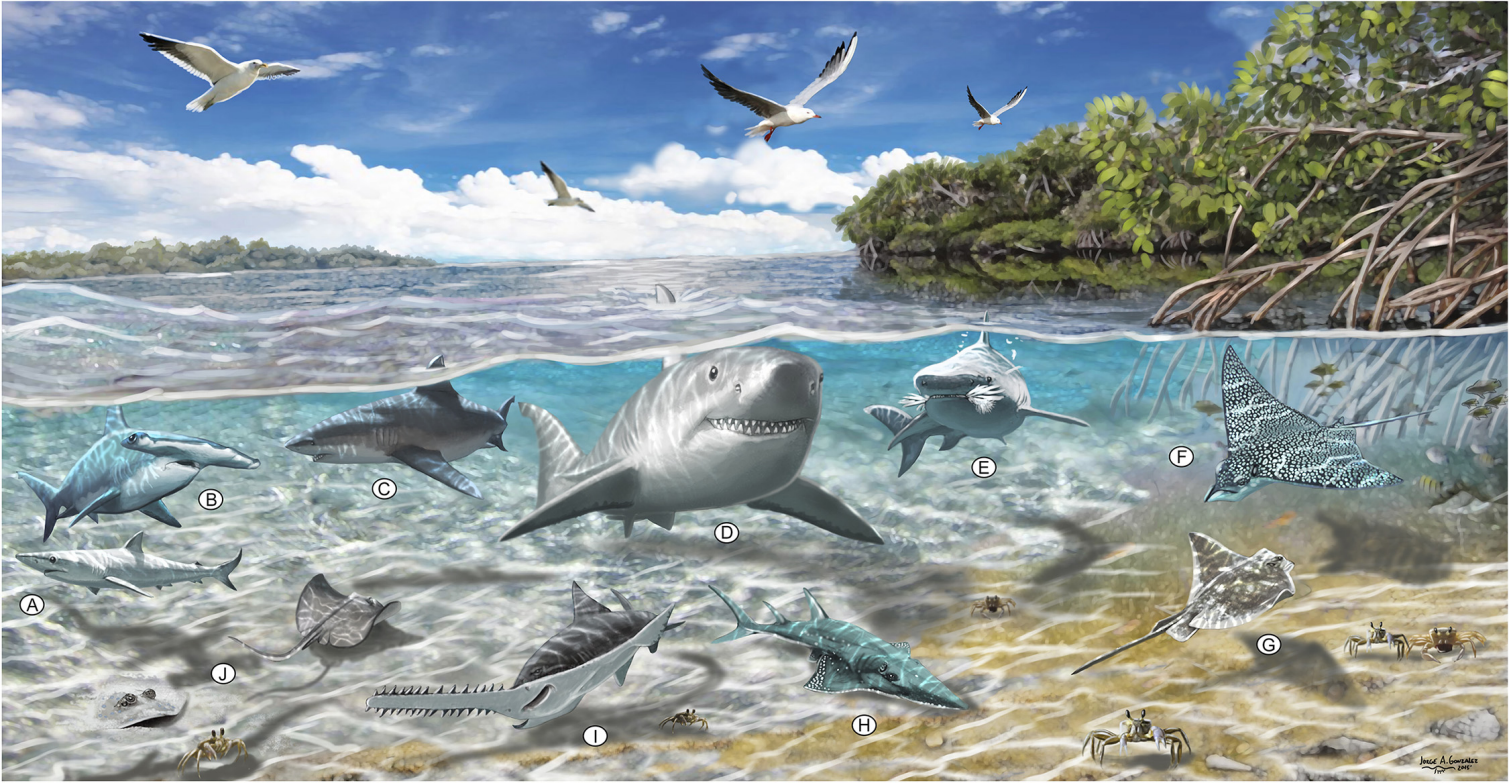
Restoration of diverse sharks and rays in coastal lagoon-estuarine at late Miocene times in Urumaco. (**A**) Sharpnose shark *Rhizoprionodon* sp., (**B**) Hammerhead shark *Sphyrna* cf. *zygaena*, (**C**) Bull shark *Carcharhinus leucas*, (**D**) “Big tooth” *Carcharocles megalodon*, (**E**) Tiger shark *Galeocerdo cuvier*, (**F**) Spotted eagle ray *Aetobatus* cf. *narinari*, (**G**) Eagle ray *Myliobatis* sp., (**H**) Guitarfish *Rhynchobatus* sp., (**I**) Sawfish *Pristis* sp., (**J**) Stingray cf. *Dasyatis*. Artwork by Jorge Gonzalez.

The elasmobranch faunistic assemblages from the Urumaco sequence (S1 Table) are distributed across a section more than 2800 m thick (Fig 2). The marine facies in the three members of the Urumaco Formation and in the Chiguaje Member (Codore Formation) have been characterized by Smith et al. [54] as shallow water paleoenviroments based on the study of foraminifera. In addition, most of the living elasmobranch taxa closely related to taxa from the Urumaco sequence prefer coastal habitats, with the exception of some species that show broader preferences (Fig 10). Extant taxa such as *Galeocerdo cuvier*, *Carcharhinus leucas*, *C*. *limbatus*, *C*. *obscurus*. *C*. *plumbeus*, *C*. *porosus*, *Negaprion brevirostris*, *Sphyrna zygaena*, *Aetobatus narinari* and representatives of the genera *Paragaleus*, *Rhizoprionodon*, *Dasyatis*, *Myliobatis*, *Rhinoptera*, *Rhynchobatus*, and *Pristis* are common inhabitants of marginal marine environments [39, 44–46]. Using the habitat preferences of living elasmobranchs (Fig 10), paleoenvironments suggested in previous studies [27], and the wide range of fossils vertebrates such as reptiles (crocodiles and turtles), mammals (cetaceans and sea cows), and especially bony fishes (S2 Table), that have been described from the whole stratigraphic sequence (including the 14 localities included in the present study) (e.g., [19, 26, 29–32, 36, 120]), we hypothesize that the fossil elasmobranch assemblages from the Urumaco sequence are associated primarily with shallow marine and estuarine environments (Fig 11) (S1 Table).

Of the fourteen localities from the Urumaco sequence with associated elasmobranch assemblages (Fig 1 and S1 Table), we suggest that the El Hatillo Norte locality (Urumaco Formation), which is characterized by a coquinoid limestone rich in marine mollusks (currently under study), and Casa el Jebe (Codore Formation) could be indicative of very shallow marine paleoenvironments. The elasmobranch assemblages at other localities of the Urumaco Formation and those of the Socorro Formation (S1 Table) are characterized by the presence of abundant remains of brackish and freshwater fishes, freshwater and marine turtles and crocodilians, and terrestrial and aquatic/semiaquatic mammals [29–32]. The association of this diverse vertebrate fauna suggests paleoenvironments associated with coastal lagoons and estuaries, especially the latter environment. Of this fossil aquatic fauna (S2 Table), at least, ten genera of freshwater fishes that includes characins, perciforms, and siluriforms (e.g., [47]), as well the turtle †*Chelus lewisi* Wood, 1976 [121], are phylogenetically close to living groups that occur today in the Orinoco and Amazon drainage systems [20, 30, 47].

The marginal marine paleoenviroments near the mouths of the rivers of the Socorro and Urumaco formations must have been subject to great variability in temperature, pH, sediment load and salinity. This is supported by the abundance in the Urumaco Formation of the foram *Ammonia parkinsoniana* which tolerates a wide range of salinities, and is a dominant taxon in low-salinity lagoons [54]. This suggests that many of the taxa that inhabited these coastal waters may have had broad salinity tolerance (Fig 10). *Galeocerdo cuvier*, *Carcharhinus limbatus*, *C*. *porosus* and *Negaprion brevirostris* are frequent in estuaries and river mouths, tolerating low levels of salinity, but do not ascend into rivers [43, 44, 46], unlike *Carcharhinus leucas* and *Pristis* species [43, 46, 47]. Batoids such as *Aetobatus narinari*, *Rhinoptera*, *Myliobatis*, and *Dasyatis* are also found in estuaries with low salinity levels [39]; *Rhinoptera bonasus* Mitchill, 1815 [122] even reproduces in the freshwater Maracaibo Lake (Venezuela) [47].

The extinct *Carcharocles megalodon* has been interpreted as a common inhabitant of tropical to warm-temperate coastal-oceanic habitats [2, 23, 24, 123], where it predated primarily cetaceans [81]. Trophic interactions between this large shark (*Carcharocles*) and marine mammals have been inferred based on remains from the early Miocene Cantaure Formation (*Carcharocles chubutensis* Ameghino, 1901[124]) and early Pliocene Paraguaná Formation (*Carcharocles megalodon*), both in Venezuela [15, 17]. However, the presence of *Carcharocles* in different marine paleoenviroments from the Neogene of Venezuela suggests that this species was a large, transient predator that may have had enough behavioral flexibility to occupy different environments, feeding on fish, turtles, cetaceans and sirenids [15, 17]. In the Urumaco sequence, especially in the Urumaco Formation, we have found isolated teeth of *Carcharocles megalodon* in marine facies and in paleoenviroments interpreted as coastal lagoons and estuaries (S1 Table). In the marine and coastal lagoon facies of the Urumaco Formation, there is no evidence of marine cetaceans; small dolphins probably related to freshwater species that inhabit the Amazon and Orinoco basins are present [30]. The occurrence of *C*. *megalodon* in the same strata as bony fishes, turtles, sirenids, and crocodiles suggests that these could possibly have been prey for this shark. At present, the presence of *C*. *megalodon* in freshwater paleoenviroments of the Urumaco sequence cannot be confirmed. Its presence in environments with variable salinity suggests that this taxon had physiological capabilities that allowed it to withstand the variations in salinity in estuarine and possibly river mouth habitats, as do some extant carcharhiniforms [43, 46].

## Conclusion

The lithologies of the Urumaco sequence are characterized by substantial variation, indicating the complexity and heterogeneity of these geologic units. Both fossils and the sedimentology document terrestrial and marine facies, including transitional paleoenviroments, and consequently a fauna tolerant to these environments. The elasmobranch fauna from the Urumaco sequence (Socorro-Urumaco-Codore formations), with almost 21 taxa, is associated principally with estuarine coastal lagoon and very shallow marine waters. The presence of elasmobranchs in association with others marine, freshwater and terrestrial vertebrates which provide seemingly contradictory signals for a palaeoenvironmental reconstruction is consistent across the larger stratigraphic sequence. This pattern is not the result of taphonomic processes, but instead proof of mixed coastal marine and fluvial-estuarine hydrographic environments during the Miocene. At the same time, the presence in the Urumaco sequence of abundant aquatic/semiaquatic vertebrates, phylogenetically close to extant groups that occur today in the Orinoco and the Amazon drainage system, support the highly debated hypothesis of a paleo-hydrographic fluvial, lacustrine or wetland complex drainage flowing along the northwestern coast of the Miocene Falcón basin into the proto-Caribbean.

## Supporting Information

S1 Appendix Geographic coordinates. (DOC)

S2 Appendix Referred fossil specimens. (DOC)

S1 Table Fossil elasmobranchs from the Urumaco sequence and paleoenvironments (XLS)

S2 Table Osteichthyan paleodiversity of the Urumaco sequence (Socorro, Urumaco and Codore formations) and the habitat of their living representatives. (XLS)
