# Supplementary material for: Sawfishes and Other Elasmobranch Assemblages from the Mio-Pliocene of the South Caribbean (Urumaco Sequence, Northwestern Venezuela)
Source: PLoS One. 2015 Oct 21;10(10):e0139230. doi: 10.1371/journal.pone.0139230 (PMC4619466; doi:10.1371/journal.pone.0139230)
Supplement: S1 Appendix — (DOC) [file pone.0139230.s001.doc]

**S1 Appendix. Geographic coordinates.**

**Socorro Formation (middle Miocene)**

-Cerro Overo (11°11'14"N, 70° 8'44"W)

- Cerro Alto (11°12'30"N, 70° 8'12"W)

**Urumaco Formation (late Miocene)**

-Sur Quebrada Bejucal (11°11'18"N, 70°15'3"W)

- Quebrada Bejucal (11°11'22"N, 70°15'20"W)

- Puente Río Urumaco (11°12'24"N, 70°15'00"W)

-Domo de Agua Blanca (11°13'25"N, 70°14'5"W)

-El Hatillo (11°14'7"N, 70°15'3"W)

-El Hatillo Norte (11°14'40"N, 70°14'44"W)

-El Mamón (11°14'1"N, 70°16'11"W)

-El Mamón Norte (11°14'27"N, 70°16'2"W)

-El Picache (11°14'26"N, 70°13'27"W)

-Corralito (11°14'57"N, 70°16'9"W)

-Tío Gregorio (11°14'42"N, 70°18'19"W)

**Codore Formation (late Miocene–Pliocene)**

-Casa El Jebe (11°15'54"N, 70°16'35"W).
