# Supplementary material for: Sawfishes and Other Elasmobranch Assemblages from the Mio-Pliocene of the South Caribbean (Urumaco Sequence, Northwestern Venezuela)
Source: PLoS One. 2015 Oct 21;10(10):e0139230. doi: 10.1371/journal.pone.0139230 (PMC4619466; doi:10.1371/journal.pone.0139230)
Supplement: S2 Appendix — (DOC) [file pone.0139230.s002.doc]

**S2 Appendix. Referred fossil specimens**.

**-Socorro Formation**: *Carcharhinus* spp. [5 teeth (AMU-CURS-630)]; *Negaprion brevirostris* [1 tooth (AMU-CURS-630)]; *Aetobatus* cf. *narinari* [1 teeth (AMU-CURS-323)]; *Myliobatis* sp. [11 teeth (AMU-CURS-322)]; *Rhinoptera* sp. [2 teeth (AMU-CURS-)]; *Pristis* sp. [ 2 rostra (AMU-CURS-241 and AMU-CURS-639)].

**-Urumaco Formation**: *Carcharocles megalodon* [15 teeth (AMU-CURS-117, 324, 330, 338, 339, 455 and 605; UNEFM-CIAAP-359, 360, 1225, 1226 and 1292; UNEFM-PF-338)]; *Hemipristis serra* [16 teeth (AMU-CURS-331, 332-337 and 362)]; *Paragaleus* sp. [1 tooth (AMU-CURS-640)]; *Galeocerdo cuvier* [2 teeth (AMU-CURS-341 and UNEFM-PF-408)]; *Rhizoprionodon* sp. [11 teeth (AMU-CURS-478, 479-481, 485, 497 and 498)]; *Carcharhinus caquetius* sp. nov. [5 teeth (AMU-CURS-477 and 499)]; *Carcharhinus leucas* [23 teeth (AMU-CURS-355, 360, 363, 364, 368, 458-462)]; *Carcharhinus limbatus* [2 teeth (AMU-CURS-456 and 457)]; *Carcharhinus obscurus* [20 teeth (AMU-CURS-356, 361, 367, 369-371, 463-467)]; *Carcharhinus porosus* [4 teeth (AMU-CURS-472, 473 and 590)]; *Carcharhinus* spp. [77 teeth (AMU-CURS-60, 358, 359, 365, 366, 373-375, 471, 486 and 629)]; *Negaprion brevirostris* [20 teeth (AMU-CURS-342, 343-349, 372, 468-470, 616, 617 and 1039)]; *Sphyrna* cf. *zygaena* [2 teeth (AMU-CURS-474; AMU-CURS-474)]; *Sphyrna* sp. [1 tooth (AMU-CURS-477)]; *Dasyatis* spp. [9 teeth (AMU-CURS-493, 495, 496 and 589)]; Dasyatidae indet. [1 tooth (AMU-CURS-591)]; *Aetobatus* cf. *narinari* [39 teeth (AMU-CURS-272, 273, 275, 276, 308, 311, 313, 318, 329, 488, and 490; UNEFM-CIAAP-1282 and 1288)]; *Myliobatis* sp. [692 teeth (AMU-CURS-59, 61, 260, 262-269, 271, 274, 277, 279, 281-283, 285, 287, 289, 303-307, 312, 314-316, 319, 326, 434, 435, 487, 615 and 618)]; *Rhinoptera* sp. [31 teeth (AMU-CURS-270, 278, 284, 286, 288, 309, 310, 317, 320, 328, 489 and 491)]; Myliobatiformes indet. [5 caudal spines (AMU-CURS-290 and 492)]; *Rhynchobatus* sp. [3 teeth (AMU-CURS-482, 483 and 628)]; *Pristis* sp. [22 rostra (AMU-CURS-23, 40, 41, 43, 45, 102, 107, 235-240, 242, 245, 251 and 376; MCNC-45-72-V, 93-72-V, 99-72-V, 154-72-V and 155-72-V), 41 rostral spines (AMU-CURS-242-244, 246,-251, 327 and 1065), and 4 bucal teeth (AMU-CURS-484)].

**-Codore Formation**: *Carcharocles megalodon* [3 teeth (AMU-CURS-599; UNEFM-PF-351)]; *Hemipristis serra* [3 teeth (AMU-CURS-623)]; *Galeocerdo cuvier* [2 teeth (AMU-CURS-625)]; *Rhizoprionodon* sp. [3 teeth (AMU-CURS-635)]; *Carcharhinus leucas* [5 teeth (AMU-CURS-622)]; *Carcharhinus plumbeus* [4 teeth (AMU-CURS-624)]; *Carcharhinus porosus* [4 teeth (AMU-CURS-632)]; *Negaprion brevirostris* [4 teeth (AMU-CURS-626)]; *Dasyatis* spp. [7 teeth (AMU-CURS-636)]; *Aetobatus* cf. *narinari* [3 fragmented dental plates (AMU-CURS-598), and 14 teeth (AMU-CURS-614)]; *Myliobatis* sp. [95 teeth (AMU-CURS-619)]; *Rhinoptera* sp. [17 teeth (AMU-CURS-621)]; Myliobatiformes indet. [1 caudal spine (AMU-CURS-634)]; cf. *Rhynchobatus* [2 teeth (AMU-CURS-638)]; *Pristis* sp. [1 rostral spine (AMU-CURS-620)].
